# High-Density Genetic Linkage Map Construction and QTL Mapping of Grain Shape and Size in the Wheat Population Yanda1817 × Beinong6

**DOI:** 10.1371/journal.pone.0118144

**Published:** 2015-02-12

**Authors:** Qiu-Hong Wu, Yong-Xing Chen, Sheng-Hui Zhou, Lin Fu, Jiao-Jiao Chen, Yao Xiao, Dong Zhang, Shu-Hong Ouyang, Xiao-Jie Zhao, Yu Cui, De-Yun Zhang, Yong Liang, Zhen-Zhong Wang, Jing-Zhong Xie, Jin-Xia Qin, Guo-Xin Wang, De-Lin Li, Yin-Lian Huang, Mei-Hua Yu, Ping Lu, Li-Li Wang, Ling Wang, Hao Wang, Chen Dang, Jie Li, Yan Zhang, Hui-Ru Peng, Cheng-Guo Yuan, Ming-Shan You, Qi-Xin Sun, Ji-Rui Wang, Li-Xin Wang, Ming-Cheng Luo, Jun Han, Zhi-Yong Liu

**Affiliations:** 1 State Key Laboratory for Agrobiotechnology / Department of Plant Genetics & Breeding, China Agricultural University, Beijing 100193, China; 2 Department of Plant Sciences, University of California at Davis, Davis 95616, United States of America; 3 Triticeae Research Institute, Sichuan Agricultural University, Wenjiang, Chengdu, Sichuan 611130, China; 4 Beijing Academy of Agriculture and Forestry Sciences, Beijing 100197, China; 5 Beijing University of Agriculture, Beijing 102206, China; Nanjing Forestry University, CHINA

## Abstract

High-density genetic linkage maps are necessary for precisely mapping quantitative trait loci (QTLs) controlling grain shape and size in wheat. By applying the Infinium iSelect 9K SNP assay, we have constructed a high-density genetic linkage map with 269 F _8_ recombinant inbred lines (RILs) developed between a Chinese cornerstone wheat breeding parental line Yanda1817 and a high-yielding line Beinong6. The map contains 2431 SNPs and 128 SSR & EST-SSR markers in a total coverage of 3213.2 cM with an average interval of 1.26 cM per marker. Eighty-eight QTLs for thousand-grain weight (TGW), grain length (GL), grain width (GW) and grain thickness (GT) were detected in nine ecological environments (Beijing, Shijiazhuang and Kaifeng) during five years between 2010–2014 by inclusive composite interval mapping (ICIM) (LOD≥2.5). Among which, 17 QTLs for TGW were mapped on chromosomes 1A, 1B, 2A, 2B, 3A, 3B, 3D, 4A, 4D, 5A, 5B and 6B with phenotypic variations ranging from 2.62% to 12.08%. Four stable QTLs for TGW could be detected in five and seven environments, respectively. Thirty-two QTLs for GL were mapped on chromosomes 1B, 1D, 2A, 2B, 2D, 3B, 3D, 4A, 4B, 4D, 5A, 5B, 6B, 7A and 7B, with phenotypic variations ranging from 2.62% to 44.39%. *QGl.cau-2A.2* can be detected in all the environments with the largest phenotypic variations, indicating that it is a major and stable QTL. For GW, 12 QTLs were identified with phenotypic variations range from 3.69% to 12.30%. We found 27 QTLs for GT with phenotypic variations ranged from 2.55% to 36.42%. In particular, QTL *QGt.cau-5A.1* with phenotypic variations of 6.82–23.59% was detected in all the nine environments. Moreover, pleiotropic effects were detected for several QTL loci responsible for grain shape and size that could serve as target regions for fine mapping and marker assisted selection in wheat breeding programs.

## Introduction

Wheat is the third highest producing cereal crop after maize and rice, and is the leading sources of vegetable protein in human food. The demand for wheat in the developing world is projected to increase by 60% by 2050 while production is expected to be affected negatively by climate change and natural resource depletion (FAO). Wheat is a staple food used to make flour for different kinds of products. Grain weight and size are the targets for breeding, not merely because they are the major components of grain yield, but also due to their impacts on milling and baking quality [1]. Moreover, grain size can partially explain the process of crop domestication [2].

Grain weight and size are complex quantitative traits controlled by a number of genes and significantly influenced by the environment. The grain weight and size can be divided into a number of components including thousand grain weight (TGW), grain length (GL), grain width (GW), and grain thickness (GT), etc. [3–7]. Previous studies have proved that TGW has high heritability values and is phenotypically the most stable yield component [8].

Monosomic and QTL analyses have been used to identify wheat genomic regions associated with grain weight and shape [3–12]. Röder et al. also reported fine genetic mapping of a grain weight QTL at the telomeric region of chromosome 7DS [13]. Up to date, no gene/QTL associated with grain shape and size has been cloned in wheat via a map-based cloning approach. However, some QTLs for grain size and weight in wheat were associated with the orthologs of rice grain traits QTLs, including *TaCwi-1A* [14], *TaSus2–2B* [15], *TaGw2–6A* [6], *TaCKX6-D1* [16], *TaSap1-A1* [17], *TaGS1–6D* [18] and *TaLsu1* [19].

Genetic dissection of grain weight and size in bread wheat, however, is greatly hampered by an enourmous genome size (~17Gb), complex genomes (allohexaploid, 2n = 42, AABBDD), and prevalence of repetitive DNA. A well-saturated genetic linkage map is a powerful tool to dissect the genetic elements responsible for grain weight and size. Both restriction fragment length polymorphisms (RFLP) and simple sequence repeats (SSR) have been used in linkage map construction, and an increasing number of QTL studies have been conducted in attempts to analysis the genetic basis of grain weight and grain size in wheat [5,7,20–27]. However, RFLP markers have shown very low levels of polymorphism between wheat cultivars, although they are co-dominant and highly reliable in nature. In contrast, SSR markers reveal a higher level polymorphism in wheat but it is very laborious to construct high-density genetic linkage maps. The need for studies of complex traits with very high density genetic linkage maps and progress in polymorphism detection and genotyping techniques has promoted the recent development of single nucletide polymorphism (SNP) markers in wheat. Meanwhile, next generation sequencing technology makes it possible to find more SNPs between wheat cultivars. The wheat Infinium iSelect 9k SNP genotyping assay was developed based on transcriptomes of 26 accessions of hexaploid wheat generated using Roche 454 and Illumina platforms [28].

In this paper, we report: 1) construction of an integrated SNP and SSR high-density genetic linkage map using Yanda1817/Beinong6 recombinant inbred lines (RILs) and an Illumina Infinium 9k SNP chip, and 2) QTL mapping of TGW, GL, GW and GT traits controlling grain shape and size in common wheat.

## Materials and Methods

### Ethics Statement

No specific permission was required for the study. The field studies did not involve endangered or protected species.

### Plant Materials and Field Trials

Yanda1817, a pure line derivative of wheat landrace Pingyao Xiaobaimai from Shanxi Province, was one of the ‘cornerstone parental’ breeding lines for the Northern China Winter Breeding Program between 1950–1960. Yanda1817 is highly tolerance to drought, winter hardiness and poor soil fertility, and has very strong tillering ability and taller plant height. More than fifty registered wheat cultivars, mostly grown in the Northern Winter Wheat Zone of China, have been generated from Yanda1817 in different breeding programs [29]. Beinong6 is a semi-dwarf high-yielding 1B/1R derivative released in the 1990s by Beijing University of Agriculture. Beinong6 consistently has larger grain size and higher kernel weight than Yanda1817. Recombinant inbred lines (RILs) of Yanda1817/Beinong6 were selected for high-density linkage map construction and QTL mapping because the RIL populations are known to be segregating widely for agronomic traits, such as plant height, yield and presence/absence of awns.

The mapping population for QTL analysis comprised 269 F_8_ to F_12_ recombinant inbred lines (RILs) derived from Yanda1817/Beinong6 by single seed descent. Compared to Yanda1817, Beinong6 shows a higher TGW and larger grain size.

Yanda1817, Beinong6 and the 269 RILs were grown in Beijing (BJ, E116.10, N40.08), Shijiazhuang (HB, E114.36, N37.38) and Kaifeng (HN, E114.23, N34.52) (S1 Fig.) from 2010–2014 at nine environments (E1–E9) for phenotype evaluations, viz., Beijing 2010 (E1), Beijing 2011 (E2), Shijiazhuang 2011 (E3), Beijing 2012 (E4), Shijiazhuang 2012 (E5), Beijing 2013 (E6), Shijiazhuang 2013 (E7), Beijing 2014 (E8) and Kaifeng 2014 (E9). Beijing (Northern Winter Wheat Zone), Shijiazhuang and Kaifeng (Yellow and Huai River Valleys Facultative Wheat Zone) represent two different wheat growing agro-climatic regions in China. The trials were performed in a randomized complete block design, and each treatment contains three replicates except for E1 and E2 which had one replicate. Each plot had two rows that were 2 m long and 25 cm wide and 30 seeds were evenly planted in each row. Field management was the same as commonly practiced in wheat production.

### Testing of Grain Traits

From the center of the rows, ten representational plants were selected to harvest as samples for measuring TGW in grams, and GL, GW and GT in millimeters. The seeds were fully cleaned and dried and broken grains were removed before trait evaluations. TGW was recorded using an electronic balance to determine the average weight of two (E1, E2, E3, E8, E9) or three (E4, E5, E6, E7) independent samples of 100 grains. GL, GW and GT were measured for 10 random grains from each RIL for each replication using vernier calipers. Trait values of each year-location combination (defined as one environment) were used for QTL analysis.

### Statistical Analysis

The broad sense heritability (H = VG/VP) of each trait was estimated from the components of variance from ANOVA. The correlation coefficients (r) between pairs of all four traits were calculated using SPSS. 20.

### DNA Extraction

Genomic DNA was extracted from two week old leaf tissue of Yanda1817, Beinong6 and each RIL using the cetyltrimethyl ammonium bromide (CTAB) method [30]. DNA was quantified using 1% agarose gel electrophoresis with λ DNA as the standard.

### SSR and EST-SSR Genotyping

Genomic SSR and EST-SSR markers (*Xcau*) were screened for polymorphisms between Yada1817 and Beinong6. Primer sequences for the Beltsville Agricultural Research Center (BARC), Gatersleben wheat microsatellite (GWM), Wheat Microsatellite Consortium (WMC), INRA Clermont-Ferrand (CFA, CFD) and Gatersleben D-genome microsatellite (GDM) were obtained from the Grain Genes website (http://wheat.pw.usda.gov/GG2/index.shtml) and public available information [31–33]. EST-SSR markers were developed according to flanking sequences of microsatellite motifs in wheat ESTs deposited in public EST databases. The polymorphic markers were used to genotype the RIL population. The PCR reactions were performed with an ABI9700 in a total volume of 10 μL containing 10 mM Tris-Hcl, pH 7.5, 50 mM MgCl_2,_ 0.2 mM dNTP, 25 ng of each primer, 0.75 U of Taq polymerase, and 50 ng of genomic DNA as the template. After an initial denaturing step for 5 min at 94°C, 35 cycles were performed for 45 s at 94°C, 55–60°C (depending on the specific primers) for 45 s, and 72°C for 70 s, with a final extension at 72°C for 10 min. PCR products were separated in 8% non-denaturing polyacrylamide gels, visualized by silver staining and photographed.

### Infinium iSelect SNP Genotyping

A total of 9,000 SNPs were selected based on their distribution across genome and frequency in the discovery population [28]. SNP genotyping was performed on the BeadStation and iScan instruments and conducted at the Genome Center of the University of California at Davis according to the manufacturer’s protocols (Illumina). Single nucleotide polymorphism allele clustering and genotype calling was performed with GenomeStudio v2011.1 software as described in Cavanagh et al. [28]. A genotype calling algorithm was generated for bread wheat using an iterative process to account for observed shifts in SNP allele cluster positions caused by differences in the number of duplicated (homeologous and paralogous) gene copies detected between assays [28, 34].

### High Density Linkage Map Construction and QTL Analysis

The linkage map was constructed with MultiPoint software and MAPMAKER/EXP version 3.0 [35] with a minimum LOD of 3.0 and maximum recombination fraction of 0.372. The Kosambi mapping function was used to convert the recombination frequencies into centiMorgan (cM) map distance [36] and the genetic linkage maps were constructed using software Map Draw V2.1 [37]. Co-segregating markers were regarded as a polymorphic locus. QTL analysis was performed with inclusive composite interval mapping by IciMapping 3.2/4.0 based on stepwise regression of simultaneous consideration of all marker information (http://www.isbreeding.net/). The ‘Deletion’ command was used to accommodate the missing phenotypes and the step size chosen was 1.0 cM. A QTL was claimed to be significant at an LOD value of 2.5.

## Results

### Phenotypic Variation and Correlation Analysis

Field trials were conducted at Beijing, Shijiazhuang and Kaifeng under different agro-climatic conditions for five continuous years (2010–2014 in 9 total environments) to evaluate TGW, GL, GW and GT variation amongst the two parents (Yanda1817 and Beinong6) and the RIL populations. Beinong6 consistently showed higher values than Yanda1817 for all the grain traits tested in 9 environments (Table 1; S1 Table). The frequency distributions of the investigated traits reveled continuous variations and transgressive segregation in the RIL populations, suggesting that the phenotypic data of TGW, GL, GW and GT are normally distributed and that the traits are controlled by multiple loci. The heritability frequencies for TGW, GL, GW and GT are 85.58%, 95.72%, 88.22% and 91.78%, respectively, indicating that the grain shape and size are stable and are mainly under genetic control (Table 1; S1 Table).

**Table 1:** Phenotypic performance and distribution parameters for grain traits of parents and RILs in nine environments.

Trait	Env.	Yanda1817	Beinong6	RIL Min.	RIL Max.	RIL average	SD	H (%)
TGW (g)	E1	23.00	39.00	15.00	46.00	29.84	5.78	85.58
	E2	29.20	51.90	18.95	52.65	32.58	6.05	
	E3	28.60	44.08	21.68	48.48	35.27	5.19	
	E4	30.63	52.46	27.83	55.32	41.75	5.23	
	E5	26.50	46.33	20.28	49.30	34.89	4.81	
	E6	22.92	42.98	17.37	44.93	29.51	5.49	
	E7	31.83	45.03	22.27	48.63	34.10	4.35	
	E8	28.28	47.13	24.32	47.84	34.70	4.47	
	E9	31.60	52.60	22.40	54.02	37.48	5.51	
GL (mm)	E1	6.13	6.70	5.10	7.12	6.01	0.35	95.72
	E2	6.00	6.60	5.16	7.29	6.28	0.36	
	E3	5.86	6.34	5.40	6.84	6.14	0.28	
	E4	6.25	6.77	5.62	7.33	6.60	0.30	
	E5	6.15	6.54	5.51	7.17	6.28	0.29	
	E6	6.07	6.94	5.62	7.22	6.40	0.30	
	E7	6.19	6.78	5.49	7.10	6.35	0.28	
	E8	6.32	6.65	5.74	7.09	6.40	0.25	
	E9	6.05	6.98	5.62	7.14	6.38	0.27	
GW (mm)	E1	2.65	3.30	2.02	3.55	2.93	0.24	88.22
	E2	2.76	3.28	2.44	3.74	2.94	0.23	
	E3	2.67	3.11	2.62	3.54	3.08	0.16	
	E4	3.16	3.62	2.97	3.78	3.40	0.14	
	E5	2.91	3.53	2.70	3.70	3.24	0.15	
	E6	2.68	3.26	2.47	3.42	2.96	0.19	
	E7	3.05	3.66	2.72	3.68	3.19	0.17	
	E8	3.04	3.33	2.75	4.06	3.18	0.16	
	E9	3.04	3.56	2.74	3.89	3.26	0.19	
GT (mm)	E1	2.45	3.06	2.10	3.27	2.70	0.22	91.78
	E2	2.56	3.11	1.50	3.48	2.65	0.25	
	E3	2.38	2.73	2.30	3.28	2.76	0.18	
	E4	2.71	3.25	2.52	3.60	3.04	0.18	
	E5	2.53	3.20	2.36	3.46	2.85	0.19	
	E6	2.48	3.10	2.21	3.18	2.71	0.17	
	E7	2.61	3.26	2.24	3.26	2.73	0.17	
	E8	2.65	2.95	2.28	3.20	2.73	0.15	
	E9	2.57	3.18	2.35	3.41	2.81	0.16	

E1, E2, E3, E4, E5, E6, E7, E8 and E9 represent Beijing 2010, Beijing 2011, Shijiazhuang 2011, Beijing 2012, Shijiazhuang 2012, Beijing 2013, Shijiazhuang 2013, Beijing 2014 and Kaifeng 2014, respectively.

Correlation coefficients (r) among the TGW, GL, GW and GT traits in different environments were calculated. All the four traits showed significant positive correlations with each other (significant at P = 0.01) (Table 2). The highest positive correlation was observed between TGW and GW (r = 0.796), followed by TGW and GT (r = 0.761). The correlation between GL and GW was very weak (r = 0.204), as well as between GL and GT (r = 0.210).

**Table 2:** Correlation coefficients between TGW, KL, GW, and GT in the RIL population in different environments.

Env.	Year	Location	TGW-GL	TGW-GW	TGW-GT	GL-GW	GL-GT	GW-GT
E1	2010	Beijing	0.499**	0.629**	0.572**	0.422**	0.434**	0.768**
E2	2011	Beijing	0.497**	0.730**	0.733**	0.399**	0.508**	0.742**
E3	2011	Shijiazhuang	0.430**	0.755**	0.787**	0.172**	0.200**	0.635**
E4	2012	Beijing	0.634**	0.642**	0.696**	0.222**	0.207**	0.408**
E5	2012	Shijiazhuang	0.533**	0.755**	0.719**	0.256**	0.156*	0.535**
E6	2013	Beijing	0.570**	0.751**	0.696**	0.237**	0.291**	0.515**
E7	2013	Shijiazhuang	0.463**	0.666**	0.566**	0.153*	0.131*	0.649**
E8	2014	Beijing	0.392**	0.456**	0.596**	0.034	0.120*	0.574**
E9	2014	Kaifeng	0.607**	0.757**	0.682**	0.307**	0.398**	0.590**
Average			0.553**	0.796**	0.761**	0.204**	0.210**	0.595**

* and ** indicate significance levels at the P = 0.05 and 0.01 (2-tailed), respectively.

### Genetic Linkage Map Construction

Out of 500 genomic SSR and EST-SSR primer pairs screened, 150 polymorphic markers were selected for RIL genotyping. Out of 8632 designated and validated SNPs in the 9k Infinium chip, 2873 SNPs were polymorphic between the parental lines Yanda1817 and Beinong6, as well as the RIL populations. Based on the 90K SNP consensus map [34] and after removing ambiguous and unlinked markers, the final genetic linkage map consists of 128 SSR, EST-SSR and 2431 SNP markers (mapped in 1062 polymorphic loci) that covered all the 21 wheat chromosomes (Table 3; S2 Table). Chromosomes 4A, 7B and 7D were integrated by two linkage groups, respectively. The entire map spaned 3213.2 cM including nine gaps (>30cM) distributed on chromosomes 1D, 2D, 3A, 3D, 6A and 7D. However, the number of markers on each chromosome was uneven, ranging from 5 on 4D to 329 on 5B. The genetic coverage of each chromosome varied from 19.1 cM (4D) to 292.9 cM (5A). All together, the markers mapping on the B genome (1301) were greatly more than those on the A genome (1093), and considerably fewer markers (165) mapped on the D genome. Only the long arm was mapped for chromosome 1B which is consistent with the fact that Beinong6 is a 1BL/1RS translocation line (data not shown). However, only a 41.3 cM genetic coverage was found for the centromere region of chromosome 1AL, and this was far below the aveage coverage of the A genome chromosomes (Table 3; S2 Table). No polymorphic SNPs was identified between Yanda1817 and Beinong6 for chromosome 1AS and the distal region of 1AL after checking the SNP mapping data [28, 34]. The possible reason for this may be that the chromosome regions of 1AS and the distal regions of 1AL have the same genetic backgrounds between Yanda1817 and Beinong6.

**Table 3:** Distribution of markers and marker density across chromosomes in the common wheat map developed in Yanda1817 × Beinong6 RILs population.

Chromosome	No. of markers	No. of loci	Map distance (cM)	Map density (cM/marker)	Map density (cM/locus)
1A	57	28	41.3	0.72	1.48
2A	234	93	187.0	0.80	2.01
3A	156	60	214.2	1.37	3.57
4A	162	61	182.6	1.13	2.99
5A	219	91	292.9	1.34	3.22
6A	108	43	146.6	1.36	3.41
7A	157	74	129.3	0.82	1.75
1B	193	67	179.8	0.93	2.68
2B	230	60	173.5	0.75	2.89
3B	228	86	263.3	1.15	3.06
4B	64	32	135.4	2.12	4.23
5B	329	119	279.6	0.85	2.35
6B	159	68	120.9	0.76	1.78
7B	98	66	221.7	2.26	3.36
1D	47	27	106.9	2.27	3.96
2D	22	16	76.2	3.46	4.76
3D	10	10	178.3	17.83	17.83
4D	5	5	19.1	3.82	3.82
5D	8	8	44.3	5.54	5.54
6D	59	34	150.6	2.55	4.43
7D	14	14	69.7	4.98	4.98
A genome	1093	450	1193.9	1.09	2.65
B genome	1301	498	1374.2	1.06	2.76
D genome	165	114	645.1	3.91	5.66
Total	2559	1062	3213.2	1.26	3.03

### Marker Density

The marker density of the individual chromosomes ranged from 0.72 cM/marker for 1A to 17.83 cM/marker for 3D with an average marker density of 1.26 cM/marker in the genetic map of Yanda1817/Beinong6 (Table 3). More markers were mapped on the A and B subgenomes with a similar marker density of 1.09 and 1.06 cM/marker, while fewer markers mapped on the D subgenome which had a density of 3.91 cM/marker. Most of the gaps were found in the D genome. For example, 7 gaps (>30cM) were identified in chromsome 1D, 2D, 3D, 6D and 7D. In addition to the gaps, 40 marker clusters (≥10 makers at one locus) were spreaded over chromosomes 1A, 1B, 2A, 2B, 3A, 3B, 4A, 4B, 5A, 5B, 6A, 6B, 6D and 7A (S2 Table). The largest marker cluster was found on chromosome 5B, which contains 63 markers, while only 6 clusters mapped on chromosome 2B. To facilicate data analysis of the 2559 markers mapping in the 1062 loci, only one marker was selected from each locus for QTL mapping. The average locus density of the genetic linkage map was 3.03 cM/locus with 2.65, 2.76 and 5.66 cM/locus for the A, B and D subgenomes, respectively (Table 3).

### Segregation Distortion Regions

Of the 1062 loci mapped in the Yanda1817 / Beinong6 genetic linkage map, 328 loci (31%) demonstrated genetic distortion (Chi-Square < 0.05) in the RIL population (S3 Table). Among the segregation distortion (SD) loci, one thrid of them (32.9%) were distorted in favor of Beinong6 and two thrids (67.1%) favored Yanda1817. Thrity-eight SD regions (SDR, ≥3 SD loci) were distributed in the whole genome except for chromosomes 1D, 2A, 3D, 5D and 7D (S3 Table). Among the SDRs, 24 were found in the B subgenome and 10 were identified in the A subgenome. Two large SDRs, SDR-1B (55 locus) were skewed to Yanda1817 and SDR-7A.3 (43 locus) favored Beinong6. SDR-1B may result from the 1RS/1BL translocation in Beinong6.

### QTL Analysis

**Fig 1:**
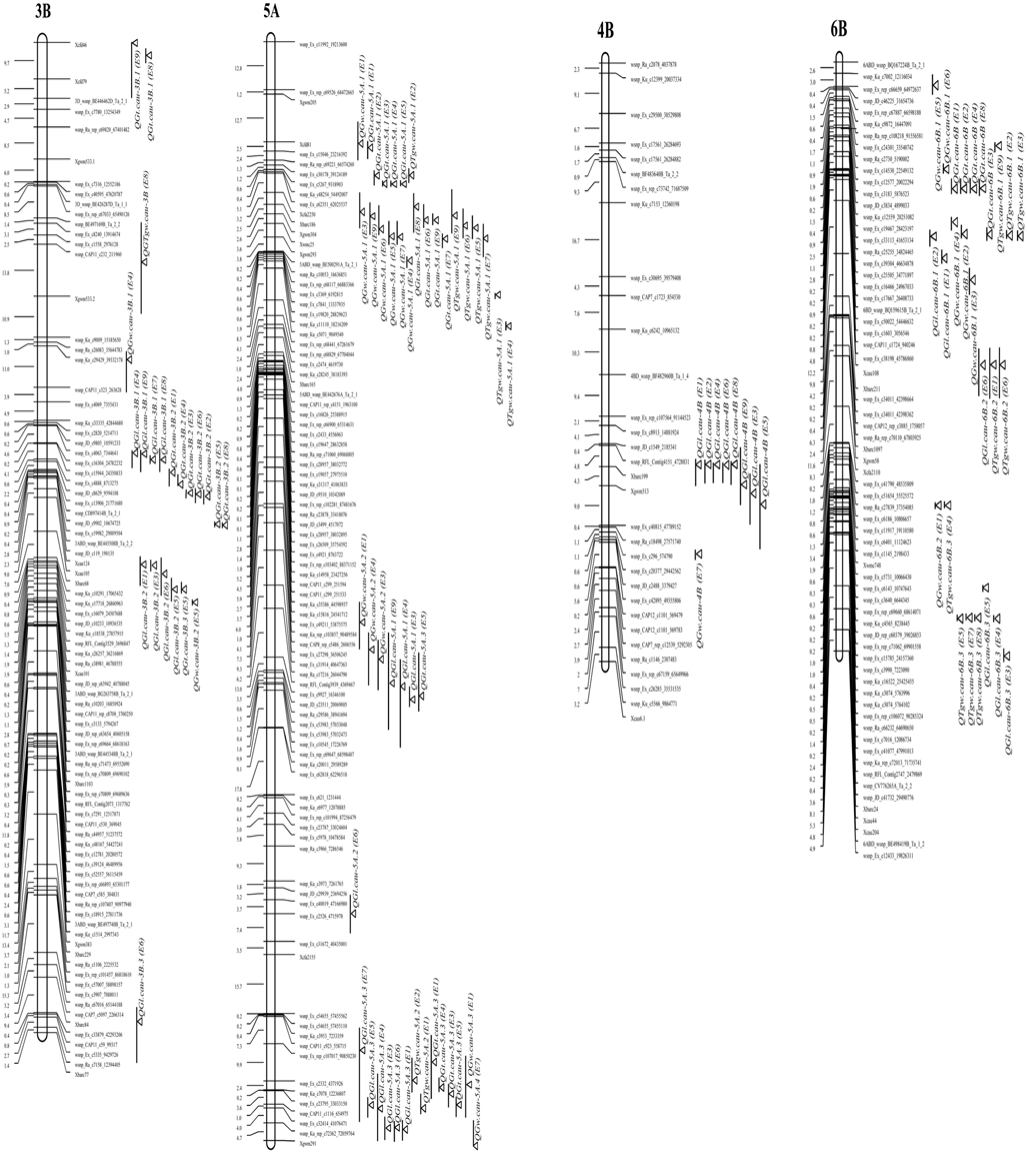
Partial pleiotropic QTL effects for TGW, GW and GT. Distribution of the detected QTLs for TGW, GL, GW and GT of the Yanda1817/Beinong6 RILs on the chromosome 3B, 4B, 5A and 6B. Supported intervals for QTL are indicated by vertical bars, the length of the bar show a one LOD confidence interval. LOD max is pointed by atriangle.

QTL mapping analyses revealed 88 putative additive QTLs for the four grain traits with phenotypic variations of single QTL ranging from 2.55% to 44.39% in different environments, and QTLs were detected on all 21 chromosomes (Table 4, 5; Fig. 1; S4, S5 Table). Chromosome 5A and 6B have a great number of identified QTLs, but chromosomes 1D, 2D, 5D, 6D and 7D have only one mapped QTL. Some QTLs appeared to be identical are closely linked even through the peaks were not at the same position. Thirty-nine individual QTLs (44.3% of all the QTLs) could be observed in at least two environments, of which 30 (76.9%) were associated with increased grain weight and size, through the Beinong6 alleles were mainly distributed on chrompsome 1B, 5A, 5B and 6B. Co-localized QTLs for different traits were also found on chromosomes 1B, 2A, 3B, 4A, 4D, 5A and 6B.

**Table 4:** Partial stable QTLs for TGW and GL detected in the Yanda1817/Beinong6 RIL population.

QTL	Env.	Chr.	Position	Left Marker	Right Marker	LOD ^a^	PVE (%) ^b^	Add ^c^	QTL Reported ^d^
*QTgw*.*cau-5A*.*1*	E2	5A	33	*wsnp_Ex_c30178_39124189*	*wsnp_Ex_c5267_9318903*	3.59	5.26	-1.36	[17], [18], [21], [35]
	E9	5A	41	*Xcfa2250*	*Xbarc186*	4.88	7.77	-1.52	
	E6	5A	43	*Xcfa2250*	*Xbarc186*	5	5.78	-1.31	
	E5	5A	44	*Xbarc186*	*Xgwm304*	4.61	6.27	-1.19	
	E7	5A	48	*Xgwm304*	*Xwmc25*	4.29	6.38	-1.09	
	E3	5A	57	*wsnp_Ex_c3369_6192815*	*wsnp_Ex_c7841_13337935*	5.13	6.25	-1.3	
	E4	5A	59	*wsnp_Ku_c11110_18216209*	*wsnp_Ku_c5071_9049540*	4.57	4.32	-1.08	
*QGl*.*cau-2A*.*2*	E3	2A	115	*wsnp_Ex_c41168_48053629*	*wsnp_Ex_c17852_26612172*	10.08	8.47	0.08	
	E4	2A	115	*wsnp_Ex_c41168_48053629*	*wsnp_Ex_c17852_26612172*	6.43	7.26	0.08	
	E5	2A	115	*wsnp_Ex_c41168_48053629*	*wsnp_Ex_c17852_26612172*	9.93	10.74	0.1	
	E6	2A	115	*wsnp_Ex_c41168_48053629*	*wsnp_Ex_c17852_26612172*	8.13	6.82	0.08	
	E1	2A	117	*wsnp_Ex_c42815_49298013*	*wsnp_Ex_rep_c103255_88258450*	4.81	5.07	0.08	
	E7	2A	117	*wsnp_Ex_c42815_49298013*	*wsnp_Ex_rep_c103255_88258450*	4.28	4.38	0.06	
	E8	2A	118	*wsnp_Ex_rep_c103255_88258450*	*wsnp_Ex_c25057_34318425*	33.7	44.39	0.17	
	E2	2A	119	*2ABD_wsnp_BG608354A_Ta_2_1*	*wsnp_Ku_c16371_25240695*	3.58	5.24	0.08	
*QGl*.*cau-4B*	E1	4B	87	*wsnp_RFL_Contig4151_4728831*	*Xbarc199*	4.2	4.43	0.07	[16]
	E2	4B	87	*wsnp_RFL_Contig4151_4728831*	*Xbarc199*	5.68	8.24	0.1	
	E4	4B	87	*wsnp_RFL_Contig4151_4728831*	*Xbarc199*	6.01	6.96	0.08	
	E6	4B	87	*wsnp_RFL_Contig4151_4728831*	*Xbarc199*	7.93	6.88	0.08	
	E8	4B	87	*wsnp_RFL_Contig4151_4728831*	*Xbarc199*	5.19	5.41	0.06	
	E9	4B	93	*Xbarc199*	*Xgwm513*	3.24	3.64	0.05	
	E3	4B	95	*Xbarc199*	*Xgwm513*	6.02	5.06	0.06	
	E5	4B	98	*Xgwm513*	*wsnp_Ex_c40815_47789152*	3.07	3.66	0.06	
	E9	2A	119	*2ABD_wsnp_BG608354A_Ta_2_1*	*wsnp_Ku_c16371_25240695*	9.13	10.04	0.09	

^a^ LOD score from the location with the underlined *P*-value

^b^ PVE (%) = phenotypic variance estimated from marker regression against phenotype

^c^ Additive effect. Positive values indicate a positive effect of Yanda1817 alleles, whereas negative values indicate the contribution of the Beinong6 allele

^d^ QTL reported by references

Seventeen QTLs for TGW were detected on chromosomes 1A, 1B, 2A, 2B, 3A, 3B, 3D, 4A, 4D, 5A, 5B and 6B with phenotypic variations ranging from 2.62% to 12.08% (Table 4; S4 Table). Seven QTLs (*QTgw*.*cau-1A*, *QTgw*.*cau-1B*, *QTgw*.*cau-4A*.*1*, *QTgw*.*cau-4D*, *QTgw*.*cau-5A*.*2*, *QTgw*.*cau-5B* and *QTgw*.*cau-6B*.*2*) were identified in two environments and five QTLs, *QTgw*.*cau-3D*.*1*, *QTgw*.*cau-6B*.*1*, *QTgw*.*cau-4A*.*2*, *QTgw*.*cau-6B*.*3* and *QTgw*.*cau-2A*, were detected in three to five environments, indicating that these QTLs are relatively stable. *QTgw*.*cau-5A*.*1*, which is the most stable QTL, could be found in seven environments except for E1 and E8. Only four QTLs mapped in one environment.

Thirty-two QTLs for GL mapped on chromosomes 1B, 1D, 2A, 2B, 2D, 3B, 3D, 4A, 4B, 4D, 5A, 5B, 6B, 7A and 7B. Each of these QTLs explaining the proportion of phenotypic variation ranged from 2.62% to 44.39% (Table 4; S4 Table). Among these QTLs, *QGl*.*cau-2A*.*2* had the highest phenotypic variation and was the most stable QTL detected in all nine environments. *QGl*.*cau-4B* was identified in eight environments, while *QGl*.*cau-5A*.*3*, *QGl*.*cau-5B*.*2* and *QGl*.*cau-7A*.*2* were present in six environments. *QGl*.*cau-1B*.*1* and *QGl*.*cau-2B*.*1* were found in five environments and two QTLs, *QGl*.*cau-3B*.*1* and *QGl*.*cau-3B*.*2*, could to be detected in four environments. Furthermore *QGl*.*cau-1B*.*3*, *QGl*.*cau-5A*.*1*, *QGl*.*cau-6B*.*1*, *QGl*.*cau-6B and QGl*.*cau-7B*.*2* were observed in two or three environments and the remaining 14 QTLs were only found in one environment. Chromosome regions contributing to GL were associated with chromosomes 2A, 2B, 3B, and 4B of Yanda1817, and chromosomes 1B, 4A, 4D, 5A, 5B, 6B, 7A and 7B of Beinong6.

Twelve chromosome regions were found to be associated with GW with phenotypic variations ranging from 3.69% to 12.30% (Table 5; S4 Table). *QGw*.*cau-5A*.*1* was found in seven environments with the strongest association with GW and this QTL explained up to 12.30% of the phenotypic variation. QTL *QGw*.*cau-6B*.*1* was detected in five environments, whereas *QGw*.*cau-5A*.*2* and *QGw*.*cau-7D* were identified in three and two environments, respectively.

**Table 5:** Partial stable QTLs for GW and GT detected in the Yanda1817/Beinong6 RIL population.

QTL	Env.	Chr.	Position	*Left Marker*	*Right Marker*	LOD ^a^	PVE (%) ^b^	Add ^c^	QTL Reported ^d^
*QGw*.*cau-5A*.*1*	E1	5A	27	*Xcfd81*	*wsnp_Ex_c15046_23216392*	3.3	4.39	-0.05	[18]
	E3	5A	39	*wsnp_Ex_c62351_62025537*	*Xcfa2250*	6.82	11.03	-0.05	
	E9	5A	41	*Xcfa2250*	*Xbarc186*	4.42	7.28	-0.05	
	E6	5A	45	*Xbarc186*	*Xgwm304*	5.92	8.63	-0.06	
	E5	5A	47	*Xgwm304*	*Xwmc25*	7.15	10.12	-0.05	
	E7	5A	47	*Xgwm304*	*Xwmc25*	7.89	12.3	-0.06	
	E4	5A	55	*Xgwm293*	*5ABD_wsnp_BE500291A_Ta_2_1*	9.13	12.03	-0.05	
*QGt*.*cau-3B*.*2*	E1	3B	107	*wsnp_Ex_c15944_24350833*	*wsnp_Ex_c4888_8713275*	6.37	7.42	-0.06	
	E4	3B	111	*wsnp_Ex_c4888_8713275*	*wsnp_JD_c8629_9594108*	9.07	8.04	-0.05	
	E3	3B	112	*wsnp_JD_c8629_9594108*	*wsnp_Ex_c13906_21771680*	3.22	2.89	-0.03	
	E6	3B	112	*wsnp_JD_c8629_9594108*	*wsnp_Ex_c13906_21771680*	4.18	3.99	-0.03	
	E2	3B	113	*wsnp_JD_c8629_9594108*	*wsnp_Ex_c13906_21771680*	4.07	6.41	-0.06	
	E5	3B	115	*wsnp_JD_c9902_10674725*	*wsnp_Ex_c19982_29009504*	27.16	36.42	-0.11	
	E8	3B	115	*wsnp_JD_c9902_10674725*	*wsnp_Ex_c19982_29009504*	4.83	6.93	-0.04	
*QGt*.*cau-5A*.*1*	E1	5A	28	*Xcfd81*	*wsnp_Ex_c15046_23216392*	8.71	10.61	-0.07	
	E2	5A	33	*wsnp_Ex_c30178_39124189*	*wsnp_Ex_c5267_9318903*	4.69	7.06	-0.06	
	E3	5A	34	*wsnp_Ex_c30178_39124192*	*wsnp_Ex_c5267_9318906*	16.47	16.72	-0.07	
	E4	5A	34	*wsnp_Ex_c30178_39124192*	*wsnp_Ex_c5267_9318906*	23.55	23.59	-0.08	
	E5	5A	34	*wsnp_Ex_c30178_39124192*	*wsnp_Ex_c5267_9318906*	11.27	12.87	-0.07	
	E8	5A	38	*wsnp_Ex_c62351_62025537*	*Xcfa2250*	4.3	6.82	-0.04	
	E6	5A	42	*Xcfa2250*	*Xbarc186*	18.26	19.85	-0.08	
	E9	5A	42	*Xcfa2250*	*Xbarc186*	10.99	17.43	-0.07	
	E7	5A	48	*Xgwm304*	*Xwmc25*	15.28	21.11	-0.08	

^a^ LOD score from the location with the underlined *P*-value

^b^ PVE (%) = phenotypic variance estimated from marker regression against phenotype

^c^ Additive effect. Positive values indicate a positive effect of Yanda1817 alleles, whereas negative values indicate the contribution of the Beinong6 allele

^d^ QTL reported by references

A total of twenty-seven QTLs for GT were identified on chromosomes 1A, 2A, 2B, 3A, 3B, 3D, 4A, 5A, 5B, 5D, 6A, 6B, 6D, 7A and 7B (Table 5; S4 Table). Among these, *QGt*.*cau-5A*.*1*, the most stable QTL locus for grain thickness and was detected in all nine environments with phenotypic variations of more than 10% in six environments. *QGt*.*cau-3B*.*2* was detected in seven environments with the highest phenotypic variation of 36.42%. Three QTLs, *QGt*.*cau-6B*, *QGt*.*cau-5A*.*3* and *QGt*.*cau-7A*, were expressed in five, four and three environments, respectively, while *QGt*.*cau-3B*.*1*, *QGt*.*cau-4A*.*1* and *QGt*.*cau-6D* were observed in two environments.

## Discussion

### A High-Density Linkage Map for QTL Mapping

Improvement of grain weight and size has always been a challenging task for breeders because it is very difficult to select complex quantitative traits such as TGW, GL, GW and GT directly. Therefore, marker-assisted selection (MAS) has been proposed as an alternative approach for indirect selection to improve the grain weight and size. With a complex and large genome, high-density marker coverage of the genome is crucial for QTL mapping in common wheat.

Previous genetic linkage maps used for QTL detection in wheat generally contained hundreds of markers, mostly AFLPs and SSRs, which were laborious and time-consuming to develop [38–42]. Here, we have constructed a high-density genetic linkage map consisting of 2559 markers (1062 polymorphic loci) that spanns 3213.2 cM covering all 21 wheat chromosomes using the recent developed Infinium iSelect 9K SNP assay intergrated with SSR and EST-SSR markers. The coverage of the Yanda1817/Beinong6 genetic linkage map is in agreement with previously reported maps in common wheat with genetic coverage from 1070 cM [5] to 4223.1 cM [41]. The B genome has the longest length and the most markers, which is also consistent with earlier reports [5,7,22,27]. In our genetic linkage map, the marker density was 1.26 cM/marker, far less than 3.7cM to 14.8cM per marker in previously reported wheat genetic linkage maps [5,7,27,40].

Another advantage of the Infinium iSelect 9K SNP assay was the high throughtput genotyping of multiply DNA samples at the same time. As the accuracy of a genetic linkage map was heavily influenced by population size, our mapping population containing 269 RILs is large enough to develop a high-density genetic linkage map with adequate genetic information for QTL analysis.

A significant phenomenon noticed in our study was the SNP marker clusters and gaps in the SNP only genetic linkage map. One possible reason may be that the SNPs were developed from the transcriptomes of 26 hexaploid wheat accessions and that most of the SNPs were derived from the gene-rich regions. Another version may be because the mapping population used for linkage map construction was developed from a cross between two Chinese wheat lines and some of the SNPs in the 9k Infinium chip were absent in the RILs. Therefore, we integrated 128 SSR and EST-SSR markers in the genetic linkage map to close some of the gaps.

### Low Level of Polymorphism in the D Genome

The polymorphic ratio of SSR and EST-SSR markers is about 30% (150/500) at the whole genome level in our mapping population, whereas a higher polymorphic ratio (33.3%, 2873/8632) was observed for SNPs. We detected relatively high SNP and SSR polymorphism levels in our mapping population and a possible reason may be due to the high divergence of the two parental lines: Yanda1817 is a Chinese landrace while Beinong6 is an advanced semi-dwarf high-yielding breeding line.

In the 3 sub-genomes, B and A have the most polymorphic markers and the D genome has the lowest number of markers. Out of 2559 polymorphic markers, only 165 markers (6.4%) mapped on the D genome, which is consistent with previous studies [33, 43–46]. The low genetic coverage of the D genome may be responsible for the low number of QTLs in the D genome.

After two polyploidization events during the common wheat evolution, the gene flow between *Ae*. *tauschii* and *T*. *aestivum* was limited to only a small population/accessions *Ae*. *stragulata* from north Iran and the southwest Caspian sea introgressed into hexaploid wheat, whereas a continuous gene flow occurred due to frequent hybridization between *T*. *aestivum* and tetraploid wheat species, and these events increased the diversity of the A and B genomes [47–49]. Increasing the genetic diversity of the D genome is still an urgent task for wheat breeders. Considering that many important genes/QTLs controlling agronomic traits were located on the D genome, additional work to increase the number and density of markers in the D genome should be considered by applying new approach like next generation sequencing (NGS).

### QTLs for Grain Shape and Size

TGW has been subjected to QTL analysis in many studies but very limited information is available for QTL mapping of GL, GW and GT in wheat. To date, QTLs for grain shape and size have been detected on almost all 21 wheat chromosomes [3,4,9–12,20,22–25,27,38,39,50–53]. Using introgression lines (ILs), Röder et al. described fine mapping of *QTgw*.*ipk-7D* associated with the microsatellite marker *Xgwm1002–7D* [13]. Due to the low coverage of chromosome 7D in our genetic map, we did not detect this QTL. QTLs for TGW were mapped to the same genetic region of chromosome 6AS by Huang et al. [39] and Sun et al. [7] using F_1_-derived doubled haploid (DH) populations and RILs, respectively. Furthermore, *TaGW2*, the ortholog of *OsGW2* in rice [54], was mapped earlier on 6AS and considered to be a candidate gene related to wheat grain development [6]. However, we did not detect any QTL on chromosome 6AS in our genetic map. In addition, due to the diversity of mapping populations, field trail conditions, and genetic coverage of the linkage maps used for QTL mapping, QTLs were often observed on different chromosome regions for grain weight and size when analyses were carried out with different phenotypic variation effects. Therefore, more refined analyses we focused on the QTLs detected at least in more than two environments.

We detected 17 QTLs for TGW and thirteen of these were found in at least two environments. In order to compare our QTL mapping data with published results, we used the integrated high-density SSR genetic linkage map [33] as a reference to anchor SSR markers and mapped QTLs (Table 4; S4, S5 Table). *QTgw*.*cau-6B*.*1* and *QTgw*.*cau-3D*.*1* were newly identified QTLs in three environments with phenotypic variation from 2.98% to 9.90%. *QTgw*.*cau-1B* was located in chromosome 1B near the *Xgwm268–1B* locus where an important QTL for TGW was previously identified using 262 accessions from a mini-core collection of Chinese wheat [26]. Similarly, *QTgw*.*cau-2A* for TGW was detected in the interval of *Xgwm249-Xgwm473* on chromosome 2A, which corresponds to the QTLs previously found by Sun et al. [7], Huang et al. [24] and Wu et al. [55], respectively. *QTgw*.*cau-4D* was closely linked to marker *Xcfd71* which may correspond to the TGW QTL reported by Huang et al. [39]. The QTL *QTgw*.*cau-5A*.*1* detected in two environments mapped in a position that also has been described by many researchers [22,23,26,27,40,52,53], indicating its stability and major effects. The *QTgw*.*cau-5A*.*2* was detected in two environments and located on the end of 5AL. In the same genetic region, QTLs for TGW were also reported by Mir et al. [56] with interval and association mapping and by Wu et al. [55] with a DH population genetic map. The interval of *QTgw*.*cau-5B* was described by Patil et al. [52] and is similar to the TGW QTL identified by Groos et al. [38] and Wang et al. [27]. In addition, Cui et al. [57] and Wu et al. [55] also detected QTLs for kernel weight per spike (KWPS) or TGW in same chromosome region. The *QTgw*.*cau-6B*.*2* identified in two environments may be same as that reported by Sun et al. [7].

Out of the 14 QTLs for GL that we detected in more than one environment, five were described in previous studies and the remaining nine may be new loci. QTL *QGl*.*cau-1B* present in 5 environments in our study was linked to marker *Xgwm259*. Sun et al. [7] also detected a QTL for GL that is associated with SSR marker *Xgwm140* which is closely linked with *Xgwm259*. A GL QTL reported by Gegas et al. [23] maps at the same location as *QGl*.*cau-3B*.*1* in our mapping study. By using two hexaploid wheat mapping populations, Breseghello and Sorrells [21] detected two QTLs for GL on 4B and 5B, which are close to *QGl*.*cau-4B* and *QGl*.*cau-5B*.*2* location in our study. On chromosome 7A, the detected QTL *QGl*.*cau-7A*.*2* seems to correspond with the QTL previously detected by Williams et al. [11].

Four GW QTLs located on 5A, 6B and 7D were detected in more than one environment, and among theses, only *QGw*.*cau-5A*.*1* was previously described [23].

QTL for grain thickness was rarely reported previously in wheat [10–12]. In our study, the GT QTLs, *QGt*.*cau-3B*.*2*, *QGt*.*cau-5A*.*1*, *QGt*.*cau-5A*.*3* and *QGt*.*cau-6B*, were identified in more than four environments. Due to the diversity of molecular markers, it was difficult to align and compare QTLs detected by these studies. TGW, GL and GW QTLs were also detected at the same chromosome regions, indicating possible linkage or pleiotropic effects.

### Trait Correlations and QTL Clustering

It was interesting that QTLs for grain size and shape clustered in same chromosome regions. In our study, co-localized QTLs were found on chromosomes 1B, 2A, 3B, 4A, 4D, 5A and 6B, and were especially prevalent on chromosomes 3B, 5A and 6B. Two QTL clusters were identified on chromosome 5A. One was located on the distal end of 5AS and is involved in controlling TGW, GW and GT. Another cluster on the distal end of 5AL is involved in regulating a GT QTL detected in four environments, a TGW QTL detected in two environments, a GL QTL detected in six environments, and a GW QTL was identified in only one environment. On chromosome 6B, QTL clusters were mainly related to TGW, GW and GT. As previous described [7,23,38,50,53,58,59] this is consistent with the positive relationships between the four grain shape and size traits, especially among TGW, GW and GT. These QTL clusters for TGW, GL, GW and GT provide important information for wheat breeders to improve the grain shape and size via marker-assisted selection.

## Supporting Information

S1 Fig The three locations {Beijing, Hebei (HB), and Henan (HN)}for field trails evaluation. (PPTX)

S1 Table The phenotypic data of Yada1817, Beinong6 and the RILs in 9 environments. (XLSX)

S2 Table The original SNP and SSR mapping data of the 269 RILs. (XLSX)

S3 Table Chi-square test for segregation distortion of locus in RIL population. (XLSX)

S4 Table QTLs detected on different chromosomes. (DOCX)

S5 Table QTLs Mapping of all the chromosomes (XLSX)

## References

[1] Gupta PK, Rustgi S, Kumar N (2006) Genetic and molecular basis of grain size and grain number and its relevance to grain productivity in higher plants. Genome 49:565–571 16936836 10.1139/g06-063

[2] Pozzi C, Rossini L, Vecchietti A, Salamini F (2005) Gene and genome changes during domestication of cereals Cereal Genomics. Springer, pp 165–198

[3] Campbell KG, Bergman CJ, Gualberto DG, Anderson JA, Giroux MJ, et al (1999) Quantitative trait loci associated with kernel traits in a soft × hard wheat cross. Crop Science 39:1184–1195

[4] Dholakia B, Ammiraju J, Singh H, Lagu M, Röder M, et al (2003) Molecular marker analysis of kernel size and shape in bread wheat. Plant Breeding 122:392–395

[5] Ramya P, Chaubal A, Kulkarni K, Gupta L, Kadoo N, et al (2010) QTL mapping of 1000-kernel weight, kernel length, and kernel width in bread wheat (*Triticum aestivum* L.). J of Appl Gene 51:421–429 10.1111/j.1472-765X.2010.02910.x 21063060

[6] Su Z, Hao C, Wang L, Dong Y, Zhang X (2011) Identification and development of a functional marker of *TaGW2* associated with grain weight in bread wheat (*Triticum aestivum* L.). Theor Appl Genet 122:211–223 10.1007/s00122-010-1437-z 20838758

[7] Sun XY, Wu K, Zhao Y, Kong FM, Han GZ, et al (2009) QTL analysis of kernel shape and weight using recombinant inbred lines in wheat. Euphytica 165:615–624

[8] Giura A, Saulescu N (1996) Chromosomal location of genes controlling grain size in a large grained selection of wheat (*Triticum aestivum* L.). Euphytica 89:77–80

[9] Varshney RK, Prasad M, Roy JK, Kumar N, Singh H, et al (2000) Identification of eight chromosomes and one microsatelite marker on 1AS associated with QTL for grain weight in bread wheat. Theor Appl Genet 100:1290–1295

[10] Williams K, Munkvold J, Sorrells M (2013) Comparison of digital image analysis using elliptic fourier descriptors and major dimensions to phenotype seed shape in hexaploid wheat (*Triticum aestivum* L.). Euphytica 190:99–116

[11] Williams K, Sorrells M (2014) Three-dimensional seed size and shape QTL in hexaploid wheat (*Triticum aestivum* L.) populations. Crop Sci 54:98–110

[12] Rasheed A, Xia XC, Ogbonnaya F, Mahmood T, Zhang ZW, et al (2014) Genome-wide association for grain morphology in synthetic hexaploid wheats using digital imaging analysis. BMC Plant Biology 14:128 10.1186/1471-2229-14-128 24884376 PMC4057600

[13] Röder MS, Huang XQ, Rörner A (2008) Fine mapping of the region on wheat chromosome 7D controlling grain weight. Funct Integr Genomics 8:79–86 17554574 10.1007/s10142-007-0053-8

[14] Ma D, Yan J, He Z (2012) Characterization of a cell wall invertase gene *TaCwi-A1* on common wheat chromosome 2A and development of functional markers. Mol Breed 29:43–52

[15] Jiang Q, Hou J, Hao C (2011) The wheat (T. aestivum) sucrose synthase 2 gene (*TaSus2*) active in endosperm development is associated with yield traits. Funct Integr Genomics 11:49–61 10.1007/s10142-010-0188-x 20821031

[16] Zhang L, Zhao Y, Gao L, Zhao G, Zhou R, et al (2012) *TaCKX6-D1*, the ortholog of rice *OsCKX2*, is associated with grain weight in hexaploid wheat. New Phytol 195:574–584 10.1111/j.1469-8137.2012.04194.x 22670578

[17] Chang J, Zhang J, Mao X (2013) Polymorphism of *TaSAP1-A1* and its association with agronomic traits in wheat. Planta 237:1495–1508 10.1007/s00425-013-1860-x 23462884

[18] Guo Y, Sun J, Zhang G, Wang Y, Kong F, et al (2013) Haplotype, molecular marker and phenotype effects associated with mineral nutrient and grain size traits of *TaGS1a* in wheat. Field Crops Res 154:119–125.

[19] Kang G, Liu G, Peng X, Wei L, Wang C, et al (2013) Increasing the starch content and grain weight of common wheat by overexpression of the cytosolic AGPase large subunit gene. Plant Physiol Biochem 73:93–98 10.1016/j.plaphy.2013.09.003 24080395

[20] Ammiraju J, Dholakia B, Santra D, Singh H, Lagu M, et al (2001) Identification of inter simple sequence repeat (ISSR) markers associated with seed size in wheat. Theor Appl Genet 102:726–732

[21] Breseghello F, Sorrells ME (2007) QTL analysis of kernel size and shape in two hexaploid wheat mapping populations. Field Crops Research 101:172–179

[22] Cuthbert JL, Somers DJ, Brûlé-Babel AL, Brown PD, Crow GH (2008) Molecular mapping of quantitative trait loci for yield and yield components in spring wheat (Triticum aestivum L.). Theor Appl Genet 117:595–608 10.1007/s00122-008-0804-5 18516583

[23] Gegas VC, Nazari A, Griffiths S, Simmonds J, Fish L, et al (2010) A genetic framework for grain size and shape variation in wheat. The Plant Cell 22:1046–1056 10.1105/tpc.110.074153 20363770 PMC2879751

[24] Huang X, Cöster H, Ganal M, Röder M (2003) Advanced backcross QTL analysis for the identification of quantitative trait loci alleles from wild relatives of wheat (*Triticum aestivum* L.). Theor Appl Genet 106:1379–1389 12750781 10.1007/s00122-002-1179-7

[25] Kumar N, Kulwal PL, Gaur A, Tyagi AK, Khurana JP, et al (2006) QTL analysis for grain weight in common wheat. Euphytica 151:135–144

[26] Wang L, Ge H, Hao C, Dong Y, Zhang X (2012) Identifying loci influencing 1,000-kernel weight in wheat by microsatellite screening for evidence of selection during breeding. PloS ONE 7:e29432 10.1371/journal.pone.0029432 22328917 PMC3273457

[27] Wang R, Hai L, Zhang X, You G, Yan C, et al (2009) QTL mapping for grain filling rate and yield-related traits in RILs of the Chinese winter wheat population Heshangmai× Yu8679. Theor Appl Genet 118:313–325 10.1007/s00122-008-0901-5 18853131

[28] Cavanagh CR, Chao S, Wang S, Huang BE, Stephen S, et al (2013) Genome-wide comparative diversity uncovers multiple targets of selection for improvement in hexaploid wheat landraces and cultivars. Proceedings of the National Academy of Sciences USA 110:8057–8062 10.1073/pnas.1217133110 23630259 PMC3657823

[29] Zhuang Q (2003) Chinese wheat improvement and pedigree analysis. Beijing: China Agriculture Press (in Chinese)

[30] Allen G, Flores-Vergara M, Krasynanski S, Kumar S, Thompson W (2006) A modified protocol for rapid DNA isolation from plant tissues using cetyltrimethylammonium bromide. Nature Protocols 1:2320–2325 17406474 10.1038/nprot.2006.384

[31] Pestsova E, Ganal M, Röder M (2000) Isolation and mapping of microsatellite markers specific for the D genome of bread wheat. Genome 43:689–697 10984182

[32] Röder MS, Korzun V, Wendehake K, Plaschke J, Tixier M-H, et al (1998) A microsatellite map of wheat. Genetics 149:2007–2023 9691054 10.1093/genetics/149.4.2007 PMC1460256

[33] Somers DJ, Isaac P, Edwards K (2004) A high-density microsatellite consensus map for bread wheat (*Triticum aestivum* L.). Theor Appl Genet 109:1105–1114 15490101 10.1007/s00122-004-1740-7

[34] Wang S, Wong D, Forrest K, Allen A, Chao S, et al (2014) Characterization of polyploid wheat genomic diversity using a high-density 90 000 single nucleotide polymorphism array. Plant Biotechnology Journal 12, pp. 787–796 10.1111/pbi.12183 24646323 PMC4265271

[35] Lincoln L, Lander E (1993) Constructing Genetic Linkage Maps with MAPMAKER/EXP Version 3.0: A Tutorial and Reference Manual. Cambridge: Lander ES:1–9

[36] Kosambi D (1943) The estimation of map distances from recombination values. Annals of Eugenics 12:172–175

[37] Liu R, Meng J (2003) MapDraw: a microsoft excel macro for drawing genetic linkage maps based on given genetic linkage data. Hereditas (Beijing) 25:317–321 15639879

[38] Groos C, Robert N, Bervas E, Charmet G (2003) Genetic analysis of grain protein-content, grain yield and thousand-kernel weight in bread wheat. Theor Appl Genet 106:1032–1040 12671751 10.1007/s00122-002-1111-1

[39] Huang X, Cloutier S, Lycar L, Radovanovic N, Humphreys D, et al (2006) Molecular detection of QTLs for agronomic and quality traits in a doubled haploid population derived from two Canadian wheats (*Triticum aestivum* L.). Theor Appl Genet 113:753–766 16838135 10.1007/s00122-006-0346-7

[40] Sun X, Marza F, Ma H, Carver BF, Bai G (2010) Mapping quantitative trait loci for quality factors in an inter-class cross of US and Chinese wheat. Theor Appl Genet 120:1041–1051 10.1007/s00122-009-1232-x 20012855

[41] Xue S, Zhang Z, Lin F, Kong Z, Cao Y, et al (2008) A high-density intervarietal map of the wheat genome enriched with markers derived from expressed sequence tags. Theor Appl Genet 117:181–189 10.1007/s00122-008-0764-9 18437345

[42] Zanetti S, Winzeler M, Feuillet C, Keller B, Messmer M (2001) Genetic analysis of bread-making quality in wheat and spelt. Plant Breeding 120:13–19

[43] Akhunov E, Nicolet C, Dvorak J (2009) Single nucleotide polymorphism genotyping in polyploid wheat with the Illumina GoldenGate assay. Theor Appl Genet 119:507–517 10.1007/s00122-009-1059-5 19449174 PMC2715469

[44] Bérard A, Le Paslier MC, Dardevet M, Exbrayat-Vinson F, Bonnin I, et al (2009) High-throughput single nucleotide polymorphism genotyping in wheat (*Triticum* spp.). Plant Biotechnology Journal 7:364–374 10.1111/j.1467-7652.2009.00404.x 19379285

[45] Chao S, Zhang W, Akhunov E, Sherman J, Ma Y, et al (2009) Analysis of gene-derived SNP marker polymorphism in US wheat (*Triticum aestivum* L.) cultivars. Molecular Breeding 23:23–33

[46] Dvorak J, Akhunov ED (2005) Tempos of gene locus deletions and duplications and their relationship to recombination rate during diploid and polyploid evolution in the *Aegilops*-*Triticum* alliance. Genetics 171:323–332 15996988 10.1534/genetics.105.041632 PMC1456522

[47] Dubcovsky J, Dvorak J (2007) Genome plasticity a key factor in the success of polyploid wheat under domestication. Science 316:1862–1866 17600208 10.1126/science.1143986 PMC4737438

[48] Luo MC, Gu YQ, You FM, Deal KR, Ma YQ, et al (2013) A 4-gigabase physical map unlocks the structure and evolution of the complex genome of *Aegilops tauschii*, the wheat D-genome progenitor. PNAS 110: 7940–7945 10.1073/pnas.1219082110 23610408 PMC3651469

[49] Wang JR, Luo MC, Chen ZX, You FM, Wei YM, et al (2013) *Aegilops tauschii* single nucleotide polymorphisms shed light on the origins of wheat D-genome genetic diversity and pinpoint the geographic origin of hexaploid wheat. New phytologist 198: 925–937 10.1111/nph.12164 23374069

[50] Quarrie S, Steed A, Calestani C, Semikhodskii A, Lebreton C, et al (2005) A high-density genetic map of hexaploid wheat (*Triticum aestivum* L.) from the cross Chinese Spring× SQ1 and its use to compare QTLs for grain yield across a range of environments. Theor Appl Genet 110:865–880 15719212 10.1007/s00122-004-1902-7

[51] Li S, Jia J, Wei X, Zhang X, Li L, et al (2007) A intervarietal genetic map and QTL analysis for yield traits in wheat. Molecular Breeding 20:167–178

[52] Patil R, Tamhankar S, Oak M, Raut A, Honrao B, et al (2013) Mapping of QTL for agronomic traits and kernel characters in durum wheat (*Triticum durum* Desf.). Euphytica 190:117–129

[53] Paux E, Sourdille P, Mackay I, Feuillet C (2012) Sequence-based marker development in wheat: advances and applications to breeding. Biotechnology Advances 30:1071–1088 10.1016/j.biotechadv.2011.09.015 21989506

[54] Song XJ, Huang W, Shi M, Zhu MZ, Lin HX (2007) A QTL for rice kernel width and weight encodes a previously unknown RING-type E3 ubiquitin ligase. Nat Genet 39:623–630 17417637 10.1038/ng2014

[55] Wu X, Chang X, Jing R (2012) Genetic insight into yield-associated traits of wheat grown in multiple rain-fed environments. PloS ONE 7:e31249 10.1371/journal.pone.0031249 22363596 PMC3281929

[56] Mir R, Kumar N, Jaiswal V, Girdharwal N, Prasad M, et al (2012) Genetic dissection of grain weight in bread wheat through quantitative trait locus interval and association mapping. Molecular Breeding 29:963–972

[57] Cui F, Zhao C, Li J, Ding A, Li X, et al (2013) Kernel weight per spike: what contributes to it at the individual QTL level? Molecular Breeding 31:265–278

[58] Peng J, Ronin Y, Fahima T, Röder MS, Li Y, et al (2003) Domestication quantitative trait loci in *Triticum dicoccoides*, the progenitor of wheat. Proceedings of the National Academy of Sciences USA 100:2489–2494 12604784 10.1073/pnas.252763199 PMC151368

[59] Marza F, Bai GH, Carver B, Zhou WC (2006) Quantitative trait loci for yield and related traits in the wheat population Ning7840 × Clark. Theor Appl Genet 112:688–698 16369760 10.1007/s00122-005-0172-3

