# Supplementary figures and images for: High-Density Genetic Linkage Map Construction and QTL Mapping of Grain Shape and Size in the Wheat Population Yanda1817 × Beinong6
Source: PLoS One. 2015 Feb 12;10(2):e0118144. doi: 10.1371/journal.pone.0118144 (PMC4326355; doi:10.1371/journal.pone.0118144)

## Slide 1
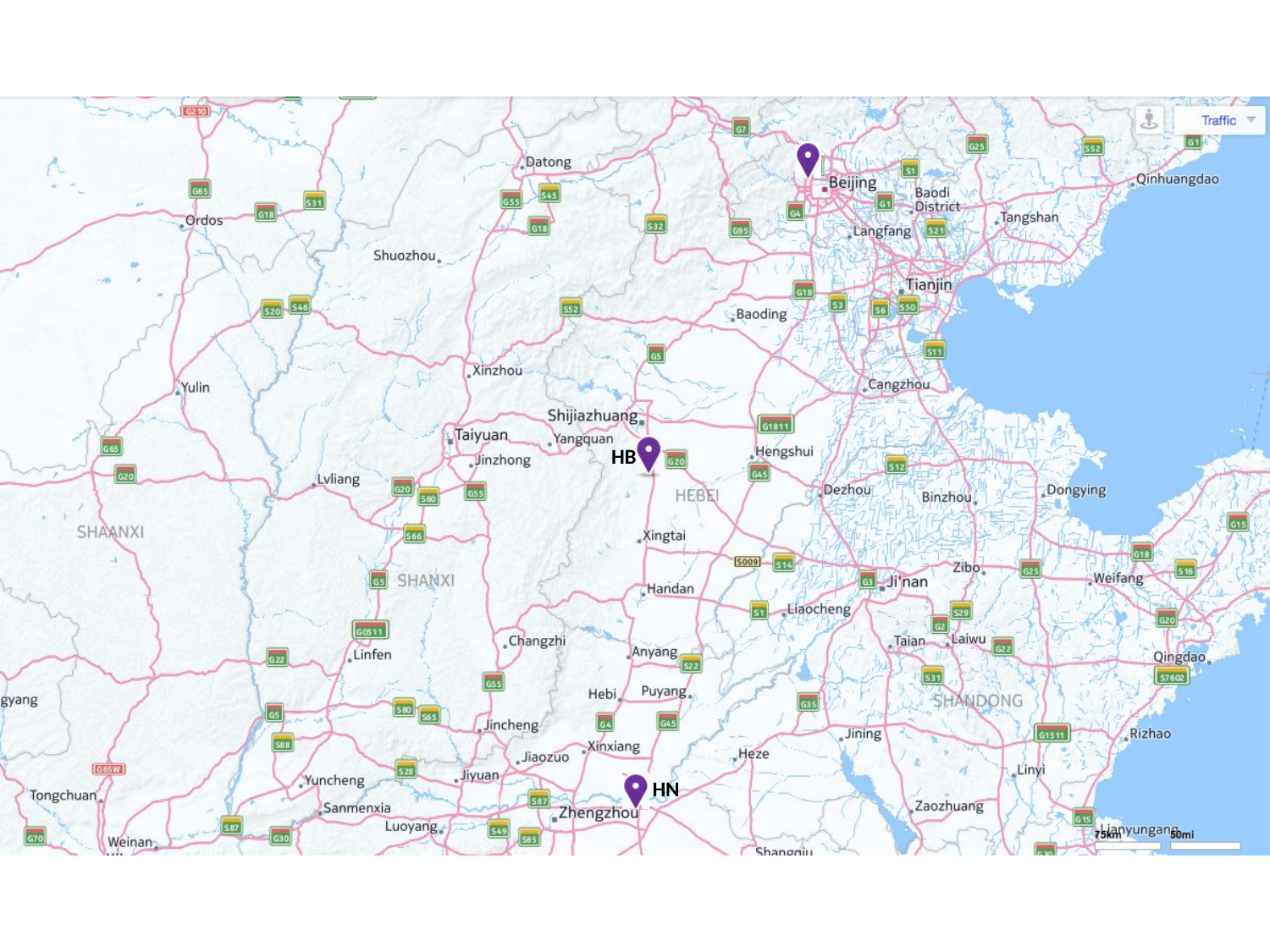

HB
HN

Supplement: S1 Fig — (PPTX) [file pone.0118144.s001.pptx]
