# Supplementary material for: High-Density Genetic Linkage Map Construction and QTL Mapping of Grain Shape and Size in the Wheat Population Yanda1817 × Beinong6
Source: PLoS One. 2015 Feb 12;10(2):e0118144. doi: 10.1371/journal.pone.0118144 (PMC4326355; doi:10.1371/journal.pone.0118144)
Supplement: S4 Table — (DOCX) [file pone.0118144.s005.docx]

Table S4 QTLs for TGW, GL, GW and GT detected in the Yanda1817/Beinong6 RIL population

| *QTL* | Env. | Chr. | Position | *Left Marker* | *Right Marker* | LOD ^a^ | PVE (%) ^b^ | Add ^c^ |
| --- | --- | --- | --- | --- | --- | --- | --- | --- |
| *QTgw.cau-1A* | E9 | 1A | 39 | *wsnp_Ex_c1845_3472790* | *wsnp_JG_c3720_1558699* | 3.14 | 4.76 | -1.19 |
|  | E6 | 1A | 41 | *wsnp_Ku_c33917_43336069* | *1AD_wsnp_BG263358A_Ta_2_1* | 3.46 | 3.90 | -1.08 |
| *QTgw.cau-1B* | E1 | 1B | 107 | *wsnp_Ex_c13878_21738866* | *Xbarc133* | 3.12 | 4.29 | -1.26 |
|  | E4 | 1B | 110 | *Xbarc133* | *wsnp_Ex_c26296_35541303* | 3.53 | 3.47 | -0.97 |
| *QTgw.cau-2A* | E3 | 2A | 118 | *wsnp_Ex_rep_c103255_88258450* | *wsnp_Ex_c25057_34318425* | 4.92 | 5.87 | 1.25 |
|  | E5 | 2A | 118 | *wsnp_Ex_rep_c103255_88258450* | *wsnp_Ex_c25057_34318425* | 5.85 | 7.59 | 1.31 |
|  | E7 | 2A | 118 | *wsnp_Ex_rep_c103255_88258450* | *wsnp_Ex_c25057_34318425* | 3.82 | 5.47 | 1.01 |
|  | E8 | 2A | 118 | *wsnp_Ex_rep_c103255_88258450* | *wsnp_Ex_c25057_34318425* | 2.66 | 3.72 | 0.81 |
|  | E4 | 2A | 119 | *2ABD_wsnp_BG608354A_Ta_2_1* | *wsnp_Ku_c16371_25240695* | 2.81 | 2.62 | 0.84 |
| *QTgw.cau-2B* | E6 | 2B | 66 | *wsnp_Ex_c14595_22634031* | *wsnp_Ex_c22018_31193171* | 3.28 | 3.65 | 1.04 |
| *QTgw.cau-3A* | E5 | 3A | 57 | *3AD_wsnp_BE443568A_Ta_2_1* | *wsnp_Ex_c25668_34932304* | 2.66 | 3.39 | -0.87 |
| *QTgw.cau-3B* | E8 | 3B | 58 | *wsnp_CAP11_c232_211960* | *Xgwm533.2* | 4.11 | 7.32 | 1.14 |
| *QTgw.cau-3D.1* | E4 | 3D | 51 | *Xgwm314* | *Xwmc656* | 3.22 | 2.98 | 0.89 |
|  | E1 | 3D | 54 | *Xwmc656* | *Xgwm645* | 3.08 | 4.71 | 1.31 |
|  | E6 | 3D | 60 | *Xwmc656* | *Xgwm645* | 4.02 | 5.47 | 1.27 |
| *QTgw.cau-3D.2* | E6 | 3D | 113 | *Xcfd55* | *wsnp_Ex_c8409_14170476* | 3.58 | 4.03 | -1.09 |
| *QTgw.cau-4A.1* | E4 | 4A | 8 | *wsnp_Ex_c5690_9994305* | *wsnp_Ex_rep_c67145_65628860* | 3.48 | 3.21 | -0.98 |
|  | E8 | 4A | 9 | *wsnp_Ex_c5690_9994305* | *wsnp_Ex_rep_c67145_65628860* | 2.67 | 4.03 | -0.89 |
| *QTgw.cau-4A.2* | E4 | 4A | 94.6 | *wsnp_Ex_c41074_47987998* | *wsnp_Ex_c41313_48161689* | 5.64 | 5.72 | -1.23 |
|  | E7 | 4A | 112.6 | *wsnp_Ex_c41313_48161689* | *wsnp_Ex_c26740_35969367* | 3.47 | 5.35 | -1.00 |
|  | E5 | 4A | 114.6 | *wsnp_Ex_c41313_48161689* | *wsnp_Ex_c26740_35969367* | 3.71 | 4.97 | -1.06 |
|  | E6 | 4A | 114.6 | *wsnp_Ex_c41313_48161689* | *wsnp_Ex_c26740_35969367* | 4.84 | 5.65 | -1.29 |
| *QTgw.cau-4D* | E3 | 4D | 0 | *wsnp_Ex_c4185_7559420* | *Xcfd71* | 4.73 | 5.63 | 1.24 |
|  | E6 | 4D | 0 | *wsnp_Ex_c4185_7559420* | *Xcfd71* | 6.52 | 7.46 | 1.50 |
| *QTgw.cau-5A.1* | E2 | 5A | 33 | *wsnp_Ex_c30178_39124189* | *wsnp_Ex_c5267_9318903* | 3.59 | 5.26 | -1.36 |
|  | E9 | 5A | 41 | *Xcfa2250* | *Xbarc186* | 4.88 | 7.77 | -1.52 |
|  | E6 | 5A | 43 | *Xcfa2250* | *Xbarc186* | 5.00 | 5.78 | -1.31 |

Table S4 continued

| *QTL* | Env. | Chr. | Position | *Left Marker* | *Right Marker* | LOD ^a^ | PVE (%) ^b^ | Add ^c^ |
| --- | --- | --- | --- | --- | --- | --- | --- | --- |
|  | E5 | 5A | 44 | *Xbarc186* | *Xgwm304* | 4.61 | 6.27 | -1.19 |
|  | E7 | 5A | 48 | *Xgwm304* | *Xwmc25* | 4.29 | 6.38 | -1.09 |
|  | E3 | 5A | 57 | *wsnp_Ex_c3369_6192815* | *wsnp_Ex_c7841_13337935* | 5.13 | 6.25 | -1.30 |
|  | E4 | 5A | 59 | *wsnp_Ku_c11110_18216209* | *wsnp_Ku_c5071_9049540* | 4.57 | 4.32 | -1.08 |
| *QTgw.cau-5A.2* | E2 | 5A | 277 | *wsnp_Ex_rep_c107017_90850230* | *wsnp_Ex_c2332_4371926* | 3.06 | 4.42 | -1.25 |
|  | E1 | 5A | 283 | *wsnp_Ex_c23795_33033150* | *wsnp_CAP11_c1116_654975* | 4.23 | 5.51 | -1.41 |
| *QTgw.cau-5B* | E3 | 5B | 62 | *wsnp_Ex_c12029_19258493* | *wsnp_Ex_c17450_26162037* | 4.66 | 5.62 | -1.22 |
|  | E4 | 5B | 62 | *wsnp_Ex_c12029_19258493* | *wsnp_Ex_c17450_26162037* | 7.21 | 6.92 | -1.36 |
| *QTgw.cau-6B.1* | E9 | 6B | 9 | *wsnp_Ex_c24301_33540742* | *wsnp_Ra_c2730_5190002* | 2.90 | 4.40 | -1.14 |
|  | E2 | 6B | 17 | *wsnp_Ex_c19467_28423197* | *wsnp_Ex_c33113_41653134* | 6.63 | 9.90 | -1.88 |
|  | E3 | 6B | 17 | *wsnp_Ex_c19467_28423197* | *wsnp_Ex_c33113_41653134* | 6.21 | 7.54 | -1.43 |
| *QTgw.cau-6B.2* | E1 | 6B | 31 | *wsnp_Ex_c38198_45786860* | *Xcau108* | 6.38 | 9.01 | -1.85 |
|  | E6 | 6B | 31 | *wsnp_Ex_c38198_45786860* | *Xcau108* | 5.29 | 6.30 | -1.39 |
| *QTgw.cau-6B.3* | E4 | 6B | 74 | *wsnp_Ex_c51654_55525572* | *wsnp_Ra_c27839_37354085* | 11.80 | 12.08 | -1.82 |
|  | E5 | 6B | 85 | *wsnp_Ex_rep_c69660_68614071* | *wsnp_Ku_c4565_8238445* | 5.15 | 6.72 | -1.24 |
|  | E7 | 6B | 85 | *wsnp_Ex_rep_c69660_68614071* | *wsnp_Ku_c4565_8238445* | 4.23 | 6.10 | -1.08 |
|  | E8 | 6B | 85 | *wsnp_Ex_rep_c69660_68614071* | *wsnp_Ku_c4565_8238445* | 5.83 | 8.44 | -1.23 |
| *QGl.cau-1B.1* | E3 | 1B | 104 | *wsnp_Ku_rep_c107952_93214466* | *wsnp_Ex_c13878_21738866* | 5.51 | 4.66 | -0.06 |
|  | E8 | 1B | 104 | *wsnp_Ku_rep_c107952_93214466* | *wsnp_Ex_c13878_21738866* | 26.89 | 34.40 | -0.15 |
|  | E1 | 1B | 105 | *wsnp_Ex_c13878_21738866* | *Xbarc133* | 8.73 | 9.68 | -0.11 |
|  | E2 | 1B | 105 | *wsnp_Ex_c13878_21738866* | *Xbarc133* | 3.92 | 5.60 | -0.09 |
|  | E7 | 1B | 105 | *wsnp_Ex_c13878_21738866* | *Xbarc133* | 9.24 | 10.05 | -0.09 |
| *QGl.cau-1B.2* | E8 | 1B | 113 | *wsnp_Ex_c26296_35541303* | *wsnp_RFL_Contig395_4387107* | 14.37 | 16.12 | 0.10 |
| *QGl.cau-1B.3* | E4 | 1B | 118 | *Xcfa2147* | *wsnp_JD_c20871_18460030* | 3.81 | 4.28 | -0.06 |
|  | E6 | 1B | 119 | *Xcfa2147* | *wsnp_JD_c20871_18460030* | 3.45 | 3.01 | -0.05 |
|  | E5 | 1B | 121 | *wsnp_JD_c20871_18460030* | *wsnp_Ex_c38849_46284348* | 3.25 | 3.45 | -0.06 |

Table S3 continued

| *QTL* | Env. | Chr. | Position | *Left Marker* | *Right Marker* | LOD ^a^ | PVE (%) ^b^ | Add ^c^ |
| --- | --- | --- | --- | --- | --- | --- | --- | --- |
| *QGl.cau-1D* | E1 | 1D | 27 | *1BD_wsnp_BE637971D_Ta_2_3* | *wsnp_CAP11_c2307_1200406* | 2.54 | 2.68 | 0.06 |
| *QGl.cau-2A.1* | E7 | 2A | 9 | *wsnp_Ex_c997_1906900* | *wsnp_Ex_c26406_35653828* | 3.12 | 3.20 | -0.05 |
| *QGl.cau-2A.2* | E3 | 2A | 115 | *wsnp_Ex_c41168_48053629* | *wsnp_Ex_c17852_26612172* | 10.08 | 8.47 | 0.08 |
|  | E4 | 2A | 115 | *wsnp_Ex_c41168_48053629* | *wsnp_Ex_c17852_26612172* | 6.43 | 7.26 | 0.08 |
|  | E5 | 2A | 115 | *wsnp_Ex_c41168_48053629* | *wsnp_Ex_c17852_26612172* | 9.93 | 10.74 | 0.10 |
|  | E6 | 2A | 115 | *wsnp_Ex_c41168_48053629* | *wsnp_Ex_c17852_26612172* | 8.13 | 6.82 | 0.08 |
|  | E1 | 2A | 117 | *wsnp_Ex_c42815_49298013* | *wsnp_Ex_rep_c103255_88258450* | 4.81 | 5.07 | 0.08 |
|  | E7 | 2A | 117 | *wsnp_Ex_c42815_49298013* | *wsnp_Ex_rep_c103255_88258450* | 4.28 | 4.38 | 0.06 |
|  | E8 | 2A | 118 | *wsnp_Ex_rep_c103255_88258450* | *wsnp_Ex_c25057_34318425* | 33.70 | 44.39 | 0.17 |
|  | E2 | 2A | 119 | *2ABD_wsnp_BG608354A_Ta_2_1* | *wsnp_Ku_c16371_25240695* | 3.58 | 5.24 | 0.08 |
|  | E9 | 2A | 119 | *2ABD_wsnp_BG608354A_Ta_2_1* | *wsnp_Ku_c16371_25240695* | 9.13 | 10.04 | 0.09 |
| *QGl.cau-2A.3* | E8 | 2A | 133 | *wsnp_Ex_c2337_4379619* | *2ABD_wsnp_BE406351A_Ta_2_2* | 18.28 | 21.15 | -0.12 |
| *QGl.cau-2B.1* | E5 | 2B | 50 | *wsnp_Ex_c35195_43389213* | *wsnp_Ex_c22010_31185837* | 7.26 | 7.74 | 0.08 |
|  | E3 | 2B | 54 | *2ABD_wsnp_BE445242B_Ta_2_1* | *wsnp_Ku_c15057_23554067* | 9.20 | 7.95 | 0.08 |
|  | E6 | 2B | 54 | *2ABD_wsnp_BE445242B_Ta_2_1* | *wsnp_Ku_c15057_23554067* | 9.87 | 8.67 | 0.09 |
|  | E9 | 2B | 54 | *2ABD_wsnp_BE445242B_Ta_2_1* | *wsnp_Ku_c15057_23554067* | 6.69 | 7.19 | 0.07 |
|  | E8 | 2B | 56 | *wsnp_Ku_c15057_23554067* | *wsnp_Ex_c27867_37030229* | 4.09 | 4.81 | 0.06 |
| *QGl.cau-2B.2* | E4 | 2B | 113 | *wsnp_Ex_c27952_37112702* | *wsnp_Ta_c36_A_1* | 3.55 | 3.96 | 0.06 |
| *QGl.cau-2D* | E9 | 2D | 20 | *Xgwm296* | *wsnp_JD_c69_109951* | 3.44 | 3.46 | -0.05 |
| *QGl.cau-3B.1* | E4 | 3B | 102 | *wsnp_Ex_c4063_7344641* | *wsnp_Ex_c16304_24782232* | 5.82 | 6.58 | 0.08 |
|  | E9 | 3B | 102 | *wsnp_Ex_c4063_7344641* | *wsnp_Ex_c16304_24782232* | 8.06 | 8.48 | 0.08 |
|  | E7 | 3B | 103 | *wsnp_Ex_c4063_7344641* | *wsnp_Ex_c16304_24782232* | 12.46 | 14.56 | 0.11 |
|  | E8 | 3B | 103 | *wsnp_Ex_c4063_7344641* | *wsnp_Ex_c16304_24782232* | 6.34 | 7.12 | 0.07 |
| *QGl.cau-3B.2* | E1 | 3B | 119 | *Xcau124* | *Xcau105* | 3.34 | 3.49 | 0.07 |
|  | E3 | 3B | 119 | *Xcau124* | *Xcau105* | 10.89 | 9.48 | 0.09 |
|  | E6 | 3B | 122 | *Xcau105* | *Xbarc68* | 4.00 | 3.47 | 0.06 |
|  | E5 | 3B | 136 | *Xbarc68* | *wsnp_Ku_c10291_17065432* | 8.40 | 9.40 | 0.09 |

Table S4 continued

| *QTL* | Env. | Chr. | Position | *Left Marker* | *Right Marker* | LOD ^a^ | PVE (%) ^b^ | Add ^c^ |
| --- | --- | --- | --- | --- | --- | --- | --- | --- |
| *QGl.cau-3B.3* | E6 | 3B | 248 | *wsnp_CAP7_c5097_2266314* | *Xbarc84* | 3.11 | 2.64 | -0.05 |
| *QGl.cau-3D.1* | E1 | 3D | 113 | *Xcfd55* | *wsnp_Ex_c8409_14170476* | 2.79 | 2.84 | -0.06 |
| *QGl.cau-3D.2* | E6 | 3D | 165 | *Xbarc342* | *Xgdm72* | 4.29 | 3.97 | -0.06 |
| *QGl.cau-4A.1* | E3 | 4A | 15 | *wsnp_Ex_c5690_9994305* | *wsnp_Ex_rep_c67145_65628860* | 3.91 | 4.18 | -0.06 |
| *QGl.cau-4A.2* | E7 | 4A | 114.6 | *wsnp_Ex_c41313_48161689* | *wsnp_Ex_c26740_35969367* | 6.87 | 7.34 | -0.08 |
| *QGl.cau-4B* | E1 | 4B | 87 | *wsnp_RFL_Contig4151_4728831* | *Xbarc199* | 4.20 | 4.43 | 0.07 |
|  | E2 | 4B | 87 | *wsnp_RFL_Contig4151_4728831* | *Xbarc199* | 5.68 | 8.24 | 0.10 |
|  | E4 | 4B | 87 | *wsnp_RFL_Contig4151_4728831* | *Xbarc199* | 6.01 | 6.96 | 0.08 |
|  | E6 | 4B | 87 | *wsnp_RFL_Contig4151_4728831* | *Xbarc199* | 7.93 | 6.88 | 0.08 |
|  | E8 | 4B | 87 | *wsnp_RFL_Contig4151_4728831* | *Xbarc199* | 5.19 | 5.41 | 0.06 |
|  | E9 | 4B | 93 | *Xbarc199* | *Xgwm513* | 3.24 | 3.64 | 0.05 |
|  | E3 | 4B | 95 | *Xbarc199* | *Xgwm513* | 6.02 | 5.06 | 0.06 |
|  | E5 | 4B | 98 | *Xgwm513* | *wsnp_Ex_c40815_47789152* | 3.07 | 3.66 | 0.06 |
| *QGl.cau-4D* | E5 | 4D | 8 | *wsnp_Ex_c4185_7559420* | *Xcfd71* | 2.81 | 3.02 | -0.05 |
| *QGl.cau-5A.1* | E9 | 5A | 156 | *wsnp_RFL_Contig3939_4369467* | *wsnp_Ex_c9927_16346100* | 3.46 | 4.79 | -0.06 |
|  | E4 | 5A | 159 | *wsnp_RFL_Contig3939_4369467* | *wsnp_Ex_c9927_16346100* | 2.56 | 3.76 | -0.06 |
|  | E3 | 5A | 167 | *wsnp_Ex_c9927_16346100* | *wsnp_JD_c23511_20069005* | 4.08 | 3.38 | -0.05 |
| *QGl.cau-5A.2* | E6 | 5A | 232 | *wsnp_Ex_c40019_47166980* | *wsnp_Ex_c2526_4715978* | 3.93 | 3.28 | -0.05 |
| *QGl.cau-5A.3* | E7 | 5A | 264 | *wsnp_CAP11_c923_558715* | *wsnp_Ex_rep_c107017_90850230* | 2.96 | 3.60 | -0.05 |
|  | E5 | 5A | 281 | *wsnp_Ex_c23795_33033150* | *wsnp_CAP11_c1116_654975* | 4.91 | 5.29 | -0.07 |
|  | E4 | 5A | 283 | *wsnp_Ex_c23795_33033150* | *wsnp_CAP11_c1116_654975* | 2.79 | 2.96 | -0.05 |
|  | E3 | 5A | 285 | *wsnp_Ex_c32414_41076471* | *wsnp_Ku_rep_c72362_72059764* | 5.43 | 4.73 | -0.06 |
|  | E6 | 5A | 285 | *wsnp_Ex_c32414_41076471* | *wsnp_Ku_rep_c72362_72059764* | 6.46 | 5.65 | -0.07 |
|  | E1 | 5A | 286 | *wsnp_Ex_c32414_41076471* | *wsnp_Ku_rep_c72362_72059764* | 7.78 | 8.96 | -0.10 |
| *QGl.cau-5B.1* | E7 | 5B | 30 | *wsnp_JD_c1845_2564931* | *5ABD_wsnp_BF201102B_Ta_2_1* | 3.85 | 4.77 | -0.06 |

Table S4 continued

| *QTL* | Env. | Chr. | Position | *Left Marker* | *Right Marker* | LOD ^a^ | PVE (%) ^b^ | Add ^c^ |
| --- | --- | --- | --- | --- | --- | --- | --- | --- |
| *QGl.cau-5B.2* | E4 | 5B | 69 | *wsnp_Ex_c10842_17637744* | *Xgwm159.1* | 7.62 | 8.95 | -0.09 |
|  | E6 | 5B | 69 | *wsnp_Ex_c10842_17637744* | *Xgwm159.1* | 5.77 | 4.97 | -0.07 |
|  | E8 | 5B | 69 | *wsnp_Ex_c10842_17637744* | *Xgwm159.1* | 2.69 | 2.75 | -0.04 |
|  | E9 | 5B | 69 | *wsnp_Ex_c10842_17637744* | *Xgwm159.1* | 4.62 | 4.78 | -0.06 |
|  | E1 | 5B | 70 | *Xgwm159.1* | *Xgwm159.2* | 4.34 | 4.52 | -0.07 |
|  | E2 | 5B | 70 | *Xgwm159.1* | *Xgwm159.2* | 2.62 | 3.70 | -0.07 |
| *QGl.cau-6B.1* | E2 | 6B | 17 | *wsnp_Ex_c19467_28423197* | *wsnp_Ex_c33113_41653134* | 2.78 | 3.85 | -0.07 |
|  | E1 | 6B | 20 | *wsnp_Ra_c25255_34824465* | *wsnp_Ex_c39304_46634878* | 5.40 | 5.86 | -0.08 |
| *QGl.cau-6B.2* | E6 | 6B | 31 | *wsnp_Ex_c38198_45786860* | *Xcau108* | 5.04 | 4.32 | -0.06 |
| *QGl.cau-6B.3* | E5 | 6B | 84 | *wsnp_Ex_c5731_10066430* | *wsnp_Ex_c6143_10747643* | 3.32 | 3.36 | -0.05 |
|  | E4 | 6B | 85 | *wsnp_Ex_rep_c69660_68614071* | *wsnp_Ku_c4565_8238445* | 4.95 | 5.54 | -0.07 |
|  | E3 | 6B | 86 | *wsnp_Ex_c15785_24157360* | *wsnp_Ex_c3990_7223090* | 6.66 | 5.45 | -0.07 |
| *QGl.cau-7A.1* | E8 | 7A | 47 | *wsnp_Ex_c20062_29096408* | *wsnp_Ex_c5341_9442913* | 5.35 | 5.55 | 0.06 |
| *QGl.cau-7A.2* | E9 | 7A | 63 | *wsnp_be500615A_Ta_1_1* | *wsnp_Ex_c28707_37811644* | 5.72 | 6.08 | -0.07 |
|  | E3 | 7A | 64 | *wsnp_Ku_rep_c71755_71490557* | *7AB_wsnp_be446380A_Ta_2_3* | 4.16 | 3.31 | -0.05 |
|  | E6 | 7A | 64 | *wsnp_Ku_rep_c71755_71490557* | *7AB_wsnp_be446380A_Ta_2_3* | 4.71 | 3.84 | -0.06 |
|  | E7 | 7A | 64 | *wsnp_Ku_rep_c71755_71490557* | *7AB_wsnp_be446380A_Ta_2_3* | 5.03 | 5.09 | -0.06 |
|  | E8 | 7A | 64 | *wsnp_Ku_rep_c71755_71490557* | *7AB_wsnp_be446380A_Ta_2_3* | 12.88 | 14.03 | -0.10 |
|  | E4 | 7A | 70 | *wsnp_JD_c14118_13933380* | *Xcau31* | 2.76 | 2.88 | -0.05 |
| *QGl.cau-7A.3* | E5 | 7A | 123 | *wsnp_Ku_c14678_23061894* | *wsnp_Ex_rep_c108367_91621570* | 6.15 | 7.12 | -0.08 |
| *QGl.cau-7B.1* | E9 | 7B | 101 | *Xgwm333* | *2ABD7ABD_wsnp_BE443010B_Ta_2_1* | 6.29 | 6.60 | -0.07 |
| *QGl.cau-7B.2* | E3 | 7B | 115 | *wsnp_RFL_Contig4753_5709032* | *wsnp_Ex_c47153_52447514* | 3.21 | 2.62 | -0.04 |
|  | E6 | 7B | 115 | *wsnp_RFL_Contig4753_5709032* | *wsnp_Ex_c47153_52447514* | 3.72 | 3.14 | -0.05 |
| *QGw.cau-2A* | E5 | 2A | 133 | *wsnp_Ex_c2337_4379619* | *2ABD_wsnp_BE406351A_Ta_2_2* | 2.98 | 3.99 | 0.03 |
| *QGw.cau-3A* | E6 | 3A | 53 | *3AD_wsnp_BQ171931A_Ta_2_1* | *wsnp_Ex_c5047_8963671* | 2.70 | 3.69 | 0.04 |
| *QGw.cau-3B.1* | E4 | 3B | 81 | *wsnp_Ku_c29429_39332178* | *wsnp_CAP11_c323_263628* | 4.07 | 4.99 | 0.03 |
| *QGw.cau-3B.2* | E5 | 3B | 138 | *wsnp_Ku_c17718_26860963* | *wsnp_Ex_c16079_24507688* | 3.23 | 4.27 | 0.03 |

Table S4 continued

| *QTL* | Env. | Chr. | Position | *Left Marker* | *Right Marker* | LOD ^a^ | PVE (%) ^b^ | Add ^c^ |
| --- | --- | --- | --- | --- | --- | --- | --- | --- |
| *QGw.cau-4B* | E7 | 4B | 106 | *wsnp_Ra_c18498_27571740* | *wsnp_Ex_c296_574790* | 2.90 | 4.14 | -0.03 |
| *QGw.cau-4D* | E6 | 4D | 1 | *wsnp_Ex_c4185_7559420* | *Xcfd71* | 3.31 | 4.87 | 0.04 |
| *QGw.cau-5A.1* | E1 | 5A | 27 | *Xcfd81* | *wsnp_Ex_c15046_23216392* | 3.30 | 4.39 | -0.05 |
|  | E3 | 5A | 39 | *wsnp_Ex_c62351_62025537* | *Xcfa2250* | 6.82 | 11.03 | -0.05 |
|  | E9 | 5A | 41 | *Xcfa2250* | *Xbarc186* | 4.42 | 7.28 | -0.05 |
|  | E6 | 5A | 45 | *Xbarc186* | *Xgwm304* | 5.92 | 8.63 | -0.06 |
|  | E5 | 5A | 47 | *Xgwm304* | *Xwmc25* | 7.15 | 10.12 | -0.05 |
|  | E7 | 5A | 47 | *Xgwm304* | *Xwmc25* | 7.89 | 12.30 | -0.06 |
|  | E4 | 5A | 55 | *Xgwm293* | *5ABD_wsnp_BE500291A_Ta_2_1* | 9.13 | 12.03 | -0.05 |
| *QGw.cau-5A.2* | E1 | 5A | 117 | *wsnp_Ku_c15816_24541712* | *wsnp_Ex_c49211_53875575* | 4.10 | 5.48 | -0.06 |
|  | E4 | 5A | 128 | *wsnp_CAP8_rep_c5486_2606556* | *wsnp_Ex_c27298_36506245* | 3.63 | 4.42 | -0.03 |
|  | E3 | 5A | 130 | *wsnp_Ex_c31914_40647363* | *wsnp_Ex_c31914_40647363* | 3.05 | 4.67 | -0.04 |
| *QGw.cau-5A.3* | E1 | 5A | 269 | *wsnp_Ex_rep_c107017_90850230* | *wsnp_Ex_c2332_4371926* | 2.73 | 4.04 | -0.05 |
|  | E7 | 5A | 292 | *wsnp_Ku_rep_c72362_72059764* | *Xgwm291* | 2.79 | 4.23 | 0.04 |
| *QGw.cau-6B.1* | E5 | 6B | 5 | *wsnp_Ku_c7002_12116034* | *wsnp_Ex_rep_c66659_64972637* | 5.57 | 7.84 | -0.04 |
|  | E6 | 6B | 12 | *wsnp_Ex_c14530_22549132* | *wsnp_Ex_c12577_20022294* | 4.75 | 6.67 | -0.05 |
|  | E4 | 6B | 16 | *wsnp_Ku_c12559_20251082* | *wsnp_Ex_c19467_28423197* | 6.19 | 7.98 | -0.04 |
|  | E2 | 6B | 17 | *wsnp_Ex_c19467_28423197* | *wsnp_Ex_c33113_41653134* | 5.37 | 9.25 | -0.07 |
|  | E3 | 6B | 23 | *wsnp_Ex_c25505_34771897* | *wsnp_Ex_c16466_24967033* | 3.70 | 5.43 | -0.04 |
| *QGw.cau-6B.2* | E1 | 6B | 74 | *wsnp_Ex_c51654_55525572* | *wsnp_Ra_c27839_37354085* | 5.61 | 7.48 | -0.07 |
| *QGw.cau-7D* | E4 | 7D | 45.2 | *Xwmc221* | *wsnp_Ex_c11813_18968198* | 3.21 | 4.70 | -0.03 |
|  | E1 | 7D | 55.2 | *wsnp_Ex_c11813_18968198* | *Xwmc94* | 3.50 | 6.38 | -0.06 |
| *QGt.cau-1A* | E1 | 1A | 34 | *Xwmc93* | *wsnp_Ex_c32590_41222878* | 2.71 | 2.98 | -0.04 |
| *QGt.cau-2A.1* | E6 | 2A | 10 | *wsnp_Ex_c997_1906900* | *wsnp_Ex_c26406_35653828* | 3.55 | 3.39 | 0.03 |
| *QGt.cau-2A.2* | E3 | 2A | 48 | *wsnp_Ex_rep_c103167_88181968* | *wsnp_Ex_rep_c103167_88182254* | 4.98 | 4.52 | 0.04 |
| *QGt.cau-2A.3* | E5 | 2A | 133 | *wsnp_Ex_c2337_4379619* | *2ABD_wsnp_BE406351A_Ta_2_2* | 3.38 | 3.68 | 0.04 |
| *QGt.cau-2B* | E3 | 2B | 121 | *wsnp_Ex_c3233_5962513* | *wsnp_Ex_rep_c67543_66165372* | 3.31 | 2.98 | 0.03 |

Table S4 continued

| *QTL* | Env. | Chr. | Position | *Left Marker* | *Right Marker* | LOD ^a^ | PVE (%) ^b^ | Add ^c^ |
| --- | --- | --- | --- | --- | --- | --- | --- | --- |
| *QGt.cau-3A.1* | E5 | 3A | 61 | *wsnp_Ex_c25668_34932304* | *wsnp_Ku_rep_c68484_67499824* | 3.15 | 3.32 | -0.03 |
| *QGt.cau-3A.2* | E1 | 3A | 115 | *wsnp_Ra_c19079_28210937* | *wsnp_Ex_c5623_9891427* | 3.54 | 4.00 | 0.04 |
| *QGt.cau-3B.1* | E9 | 3B | 0 | *Xcfd46* | *Xcfd79* | 2.68 | 3.77 | 0.03 |
|  | E8 | 3B | 2 | *Xcfd46* | *Xcfd79* | 3.97 | 6.43 | 0.04 |
| *QGt.cau-3B.2* | E1 | 3B | 107 | *wsnp_Ex_c15944_24350833* | *wsnp_Ex_c4888_8713275* | 6.37 | 7.42 | -0.06 |
|  | E4 | 3B | 111 | *wsnp_Ex_c4888_8713275* | *wsnp_JD_c8629_9594108* | 9.07 | 8.04 | -0.05 |
|  | E3 | 3B | 112 | *wsnp_JD_c8629_9594108* | *wsnp_Ex_c13906_21771680* | 3.22 | 2.89 | -0.03 |
|  | E6 | 3B | 112 | *wsnp_JD_c8629_9594108* | *wsnp_Ex_c13906_21771680* | 4.18 | 3.99 | -0.03 |
|  | E2 | 3B | 113 | *wsnp_JD_c8629_9594108* | *wsnp_Ex_c13906_21771680* | 4.07 | 6.41 | -0.06 |
|  | E5 | 3B | 115 | *wsnp_JD_c9902_10674725* | *wsnp_Ex_c19982_29009504* | 27.16 | 36.42 | -0.11 |
|  | E8 | 3B | 115 | *wsnp_JD_c9902_10674725* | *wsnp_Ex_c19982_29009504* | 4.83 | 6.93 | -0.04 |
| *QGt.cau-3B.3* | E5 | 3B | 136 | *Xbarc68* | *wsnp_Ku_c10291_17065432* | 16.48 | 20.79 | 0.08 |
| *QGt.cau-3D* | E6 | 3D | 63 | *Xgwm645* | *Xcfd55* | 4.86 | 4.62 | 0.04 |
| *QGt.cau-4A.1* | E3 | 4A | 98.6 | *wsnp_Ex_c41074_47987998* | *wsnp_Ex_c41313_48161689* | 2.78 | 3.22 | -0.03 |
|  | E9 | 4A | 106.6 | *wsnp_Ex_c41074_47987998* | *wsnp_Ex_c41313_48161689* | 2.72 | 4.38 | -0.03 |
| *QGt.cau-4A.2* | E7 | 4A | 156.6 | *wsnp_Ex_c410_808465* | *wsnp_Ex_c410_811343* | 2.79 | 3.39 | -0.03 |
| *QGt.cau-5A.1* | E1 | 5A | 28 | *Xcfd81* | *wsnp_Ex_c15046_23216392* | 8.71 | 10.61 | -0.07 |
|  | E2 | 5A | 33 | *wsnp_Ex_c30178_39124189* | *wsnp_Ex_c5267_9318903* | 4.69 | 7.06 | -0.06 |
|  | E3 | 5A | 34 | *wsnp_Ex_c30178_39124192* | *wsnp_Ex_c5267_9318906* | 16.47 | 16.72 | -0.07 |
|  | E4 | 5A | 34 | *wsnp_Ex_c30178_39124192* | *wsnp_Ex_c5267_9318906* | 23.55 | 23.59 | -0.08 |
|  | E5 | 5A | 34 | *wsnp_Ex_c30178_39124192* | *wsnp_Ex_c5267_9318906* | 11.27 | 12.87 | -0.07 |
|  | E8 | 5A | 38 | *wsnp_Ex_c62351_62025537* | *Xcfa2250* | 4.30 | 6.82 | -0.04 |
|  | E6 | 5A | 42 | *Xcfa2250* | *Xbarc186* | 18.26 | 19.85 | -0.08 |
|  | E9 | 5A | 42 | *Xcfa2250* | *Xbarc186* | 10.99 | 17.43 | -0.07 |
|  | E7 | 5A | 48 | *Xgwm304* | *Xwmc25* | 15.28 | 21.11 | -0.08 |
| *QGt.cau-5A.2* | E5 | 5A | 165 | *wsnp_Ex_c9927_16346100* | *wsnp_JD_c23511_20069005* | 5.68 | 6.51 | 0.05 |
| *QGt.cau-5A.3* | E1 | 5A | 269 | *wsnp_Ex_rep_c107017_90850230* | *wsnp_Ex_c2332_4371926* | 5.57 | 6.93 | -0.06 |

Table S4 continued

| *QTL* | Env. | Chr. | Position | *Left Marker* | *Right Marker* | LOD | PVE (%) | Add |
| --- | --- | --- | --- | --- | --- | --- | --- | --- |
|  | E4 | 5A | 279 | *wsnp_Ex_c2332_4371926* | *wsnp_Ku_c7078_12236807* | 5.82 | 5.13 | -0.04 |
|  | E3 | 5A | 280 | *wsnp_Ex_c23795_33033150* | *wsnp_CAP11_c1116_654975* | 5.80 | 5.43 | -0.04 |
|  | E5 | 5A | 282 | *wsnp_Ex_c23795_33033150* | *wsnp_CAP11_c1116_654975* | 4.65 | 5.18 | -0.04 |
| *QGt.cau-5B.1* | E1 | 5B | 18 | *Xgwm234* | *wsnp_Ex_c607_1204733* | 4.91 | 6.87 | -0.06 |
| *QGt.cau-5B.2* | E4 | 5B | 61 | *wsnp_Ex_c12029_19258493* | *wsnp_Ex_c17450_26162037* | 4.58 | 4.00 | -0.03 |
| *QGt.cau-5B.3* | E7 | 5B | 80 | *wsnp_Ex_c44230_50338772* | *wsnp_Ex_rep_c69760_68719014* | 4.62 | 5.72 | -0.04 |
| *QGt.cau-5B.4* | E3 | 5B | 103 | *wsnp_Ex_c57667_59284398* | *wsnp_Ku_c12562_20256747* | 15.49 | 15.78 | -0.07 |
| *QGt.cau-5B.5* | E3 | 5B | 115 | *wsnp_Ku_rep_c68797_67943795* | *wsnp_Ra_c26091_35652620* | 6.48 | 6.04 | 0.04 |
| *QGt.cau-5D* | E7 | 5D | 44 | *Xgwm159.3* | *Xcfd78* | 3.06 | 3.78 | 0.03 |
| *QGt.cau-6A* | E6 | 6A | 34 | *wsnp_Ra_c17221_26052231* | *wsnp_Ku_c2700_5121331* | 3.05 | 2.81 | 0.03 |
| *QGt.cau-6B* | E1 | 6B | 14 | *wsnp_Ex_c12577_20022294* | *wsnp_Ex_c3183_5876523* | 5.61 | 6.48 | -0.06 |
|  | E2 | 6B | 14 | *wsnp_Ex_c12577_20022294* | *wsnp_Ex_c3183_5876523* | 4.33 | 6.61 | -0.06 |
|  | E4 | 6B | 14 | *wsnp_Ex_c12577_20022294* | *wsnp_Ex_c3183_5876523* | 7.67 | 6.76 | -0.05 |
|  | E8 | 6B | 14 | *wsnp_Ex_c12577_20022294* | *wsnp_Ex_c3183_5876523* | 3.10 | 4.45 | -0.03 |
|  | E3 | 6B | 17 | *wsnp_Ex_c19467_28423197* | *wsnp_Ex_c33113_41653134* | 4.96 | 4.59 | -0.04 |
| *QGt.cau-6D* | E6 | 6D | 29 | *Xpsp3200* | *Xwmc671* | 2.68 | 5.87 | -0.04 |
|  | E7 | 6D | 36 | *Xpsp3200* | *Xwmc671* | 3.24 | 8.06 | -0.05 |
| *QGt.cau-7A* | E4 | 7A | 91 | *wsnp_Ex_c5177_9174930* | *wsnp_Ra_c10878_17788898* | 3.02 | 2.55 | 0.03 |
|  | E3 | 7A | 92 | *wsnp_Ku_c44600_51841068* | *wsnp_Ex_c12102_19361467* | 2.83 | 2.60 | 0.03 |
|  | E5 | 7A | 99 | *wsnp_Ex_c12102_19361467* | *wsnp_Ra_c8394_14242442* | 3.07 | 3.53 | 0.03 |
| *QGt.cau-7B* | E6 | 7B | 100 | *7ABD_wsnp_be442619B_Ta_2_1* | *Xgwm333* | 4.05 | 4.01 | -0.03 |

^a^ LOD score from the location with the underlined *P*-value; ^b^ PVE (%) = phenotypic variance estimated from marker regression against phenotype

^c^ Additive effect. Positive values indicate a positive effect of Yanda1817 alleles, whereas negative values indicate the contribution of the Beinong6 allele.
